# Hedan tablet ameliorated non‐alcoholic steatohepatitis by moderating NF‐κB and lipid metabolism‐related pathways via regulating hepatic metabolites

**DOI:** 10.1111/jcmm.18194

**Published:** 2024-03-20

**Authors:** Liying Guo, Jinyan Lei, Peng Li, Yuming Wang, Jing Wang, Taotao Song, Bo Zhu, Jianwei Jia, Jing Miao, Huantian Cui

**Affiliations:** ^1^ Department of Chinese Medicine Tianjin Second People's Hospital Tianjin China; ^2^ Graduate School Tianjin University of Traditional Chinese Medicine Tianjin China; ^3^ First School of Clinical Medicine Yunnan University of Chinese Medicine Kunming China

**Keywords:** Hedan tablet, lipid metabolism, NF‐κB pathway, non‐alcoholic steatohepatitis, untargeted metabolomic

## Abstract

Non‐alcoholic steatohepatitis (NASH) is a severe form of fatty liver disease. If not treated, it can lead to liver damage, cirrhosis and even liver cancer. However, advances in treatment have remained relatively slow, and there is thus an urgent need to develop appropriate treatments. Hedan tablet (HDP) is used to treat metabolic syndrome. However, scientific understanding of the therapeutic effect of HDP on NASH remains limited. We used HDP to treat a methionine/choline‐deficient diet‐induced model of NASH in rats to elucidate the therapeutic effects of HDP on liver injury. In addition, we used untargeted metabolomics to investigate the effects of HDP on metabolites in liver of NASH rats, and further validated its effects on inflammation and lipid metabolism following screening for potential target pathways. HDP had considerable therapeutic, anti‐oxidant, and anti‐inflammatory effects on NASH. HDP could also alter the hepatic metabolites changed by NASH. Moreover, HDP considerable moderated NF‐κB and lipid metabolism‐related pathways. The present study found that HDP had remarkable therapeutic effects in NASH rats. The therapeutic efficacy of HDP in NASH mainly associated with regulation of NF‐κB and lipid metabolism‐related pathways via arachidonic acid metabolism, glycine‐serine‐threonine metabolism, as well as steroid hormone biosynthesis.

## 1 INTRODUCTION

Non‐alcoholic fatty liver disease (NAFLD) is a widespread liver condition, affecting a large portion of the global population. In recent years, its incidence has risen to about 25%.^1^ Non‐alcoholic steatohepatitis (NASH) is a severe form of NAFLD. If not treated, it can lead to liver damage, cirrhosis and even liver cancer.^2^ Although the medical community has made steady progress in the epidemiology, pathogenesis and identification of therapeutic targets for NASH, advances in treatment have remained relatively slow, and there is thus an urgent need to develop appropriate treatments.

Traditional Chinese medicine (TCM) and the application of its active components have contributed many clinically effective therapies for the treatment of NAFLD and NASH. Xiao‐Chai‐Hu decoction has demonstrated effectiveness in treating NASH by regulating lipid metabolism and exhibiting anti‐inflammatory properties.^3^ Shaofu Zhuyu decoction ameliorated NAFLD by improving hepatic steatosis and systemic inflammation, in addition to reversing the alterations in tricarboxylic acid cycle, aromatic amino acid metabolism and pentose phosphate pathway.^4^ Qushi Huayu decoction not only protect intestinal barrier function via the MAPK pathway, thereby inhibiting intestinal leakage of liposaccharides, but also enhance the transfer of PPAR‐γ and p‐p65 to the nucleus and the reprogramming of haematopoietic stem cells in the liver, providing protection against steatosis and fibrosis.^5^, ^6^, ^7^

Hedan tablet (HDP) is composed of *Salvia miltiorrhiza* Bunge, *Crataegus pinnatifida* Bunge, *Nelumbo nucifera* Gaertn., *Senna alexandrina* var. alexandrina, and *Cullen corylifolium* (L.) Medik. A randomized double‐blind trial showed that HDP significantly improved body weight, body mass index, serum levels of LDL‐C and lipocalin, and TG/HDL‐C ratio in patients with metabolic syndrome.^8^, ^9^ However, scientific understanding of the therapeutic effect of HDP on NASH remains limited. In this study, we investigated the therapeutic effects of HDP on liver injury using a methionine/choline‐deficient (MCD) diet‐induced model of NASH in rats. In addition, untargeted metabolomics were used to investigate effects of HDP on hepatic metabolites of NASH rats, and further validated its effects on inflammation and lipid metabolism following screening for potential target pathways.

## 2 METHODS

### 2.1 Experimental animals and reagents

Male SD rats (aged 8 weeks, 250–300 g) were purchased from Beijing Huafukang Bioscience. Detailed information on reagents, kits and antibodies are provided in the supplemental material. Animal experiments were approved by Ethics Committee of Tianjin Second People's Hospital (Approval No: SL‐2022‐0314), dated 14 March 2022.

### 2.2 Animal experiments

Sixty rats were acclimated for 1 week, and then they were randomly divided into six groups: control, model, PPC, L‐HDP, M‐HDP and H‐HDP, with 10 rats per group. From Day 1 of the experiment, all groups were provided with the MCD diet^10^, ^11^, ^12^ (see supplemental materials for formulation), except for control group, which accepted a normal diet. A treatment of 62 mg/kg/d PPC was administered by gavage to the PPC group and 197, 394, and 788 mg/kg/d HDP was orally administered to the L‐HDP, M‐HDP and H‐HDP groups, respectively. Vehicle was orally administered to control and model groups in equal volume. All rats were weighed every 7 days. On Day 42, the rats were anaesthetised, blood was collected and animals were then euthanized. Serum was isolated and frozen for storage. Liver was promptly removed and weighed. Liver lobes from a consistent location were separated and fixed in a 4% paraformaldehyde or cryopreservation, while remaining liver tissues were stored frozen. All therapeutic doses were calculated according to the pharmacological body surface area method. The M‐HDP group represented the equivalent dose group for 70 kg adult humans, and the L‐HDP and H‐HDP groups represented 0.5‐fold and 2‐fold M‐HDP group doses, respectively.

### 2.3 Histological analysis

Liver fixed in 4% paraformaldehyde were processed into paraffin sections for HE staining. NASH activity score (NAS) was performed by light microscopy (E100, Nikon, Tokyo, Japan) to assess pathological changes and liver tissue inflammation as described previously.^13^ In addition, frozen tissue sections were prepared and stained with Oil Red O to observe fat accumulation within liver tissue. The formation of fat droplets and the expansion of adipocytes in liver tissue were assessed by light microscopy and quantified by average optical density (AOD).

### 2.4 Biochemical tests

Liver tissue was added to saline at a w/v ratio of 1:9. Next, the mixture was placed in an ice bath and homogenized using an ultrasonic homogenizer. The mixture was centrifuged (500× *g*, 10 min) and supernatant was collected for subsequent experiments. Biochemical indicators total cholesterol (TC), triglyceride (TG), glutathione peroxidase (GSH‐Px), malondialdehyde (MDA), superoxide dismutase (SOD), and total protein concentration of the homogenates were determined using corresponding kits. Serum alanine aminotransferase (ALT), and aspartate aminotransferase (AST) activities were also measured using corresponding kits. All procedures were performed following manufacturers' instructions.

### 2.5 ELISA

One‐step double‐antibody sandwich ELISA experiments were used to measure hepatic levels of cytokines, IL‐1β, IL‐6 and TNF‐α. Briefly, microtiter wells were coated with capture antibodies. Test samples, standards and detection antibodies were then added. After incubation and washing, a reaction substrate was added to induce colour development. Finally, the absorbance was measured, and a standard curve were constructed to calculate the corresponding concentration of the substance in each test sample.

### 2.6 RT‐qPCR

Conventional procedures were performed as previously described.^14^ Briefly, total RNA was extracted from liver tissue using Trizol and reverse translated to cDNA. Gene expression analysis was performed using a real‐time PCR detection system and the SuperReal PreMix Plus kit. The relative expression between target genes and the reference gene *Actb* was calculated using 2^−ΔΔCT^ method. Primers are detailed in the Table S1.

### 2.7 Liver tissue metabolomics

Conventional procedures were performed as previously described.^15^ First, liver tissues pulverized in liquid nitrogen were mixed with 80% methanol at a 1:5 ratio, and the samples were processed by vortexing and shaking. Next, samples were centrifuged at 4°C (15,000 × *g*, 20 min), the supernatant was diluted to 53% methanol and centrifuged again (15,000 × *g*, 20 min), and supernatant was collected for subsequent assays. An equal amount (20 μL) of supernatant was aspirated from each sample for mixing and used as quality control (QC) samples. The gradient elution program used is shown in Table S2. Details on protocols and data processing are listed in the supplemental materials section.

### 2.8 Western blot

Total protein was extracted from frozen liver tissue using RIPA lysis buffer. Next, protein concentration of samples was measured using BCA assay. The protein samples were then performed SDS‐PAGE and electroblotted onto PVDF membranes. After blocking, primary antibody and secondary antibody were used for incubation sequency. Then, fluorescence development was performed using a high‐sensitivity ECL (ChemiDocXRS+, Bio‐Rad and Hercules, CA, USA) and membrane was imaged. ImageJ 1.52a (NIH, Bethesda, MD, USA) was used to quantify band intensity.

### 2.9 Statistics

Statistical analysis was performed using SPSS 22.0 (IBM, Armonk, NY, USA). All data are expressed as mean ± standard deviation (SD). Differences among groups were compared using one‐way analysis of variance with Tukey's honest significant difference test. Statistical significance was considered at *p* < 0.05.

## 3 RESULTS

### 3.1 Therapeutic effects of HDP in NASH rats

**FIGURE 1:**
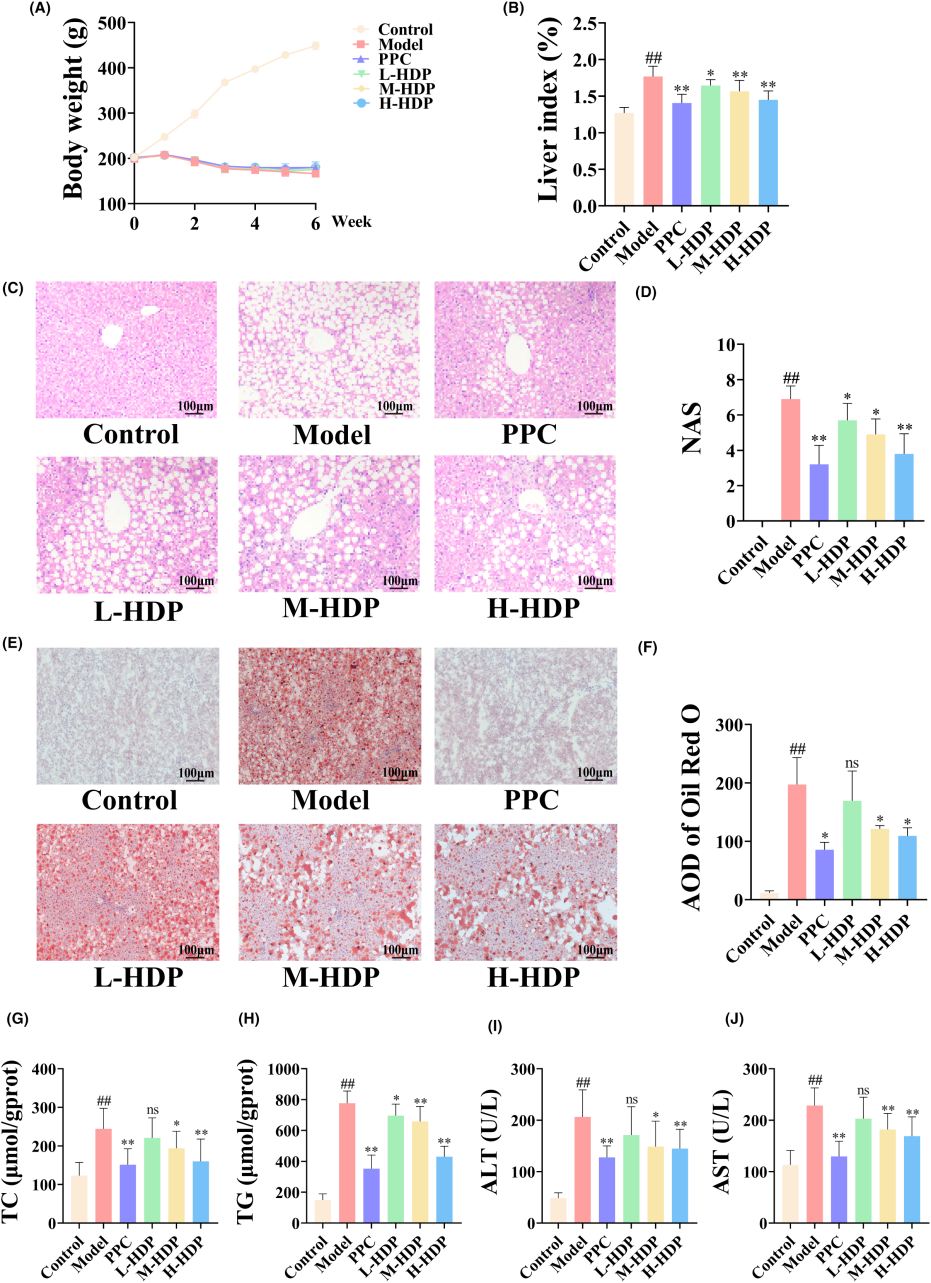
Therapeutic effects of HDP on NASH rat. After 42 days of treatment, HDP exhibited considerable therapeutic effects on NASH rat. (A) Body weight change curves of each group. (B) HDP reduced liver index of NASH rat. (C–F) HE staining and Oil Red O staining showed the alleviation of pathology and lipid accumulation in liver (200×). (G–I) Changes in hepatic lipid profile and liver function. Data are shown as means ± SD (Control, Model, IRB, L‐HDP, M‐HDP, and H‐HDP groups, *n* = 10 per group). ^##^: *p* < 0.01 versus control group; *: *p* < 0.05 and **: *p* < 0.01 versus model group.

Rats exhibited significant symptoms of NASH after receiving the MCD diet for 42 days. Compared to control group, NASH rats exhibited a significant decrease in body weight, and their liver index was increased (Figure 1A,B). PPC or HDP treatment did not improve the body weight of NASH rats, but reduced the liver index to varying degrees. HE results show that the hepatic plates of control rats were well‐organized, displaying normal structures and no significant abnormalities. The livers of rats in model group displayed significant vacuolar steatosis and extensive inflammatory cell infiltration. However, liver of rats in the PPC and HDP groups showed a considerable reduction in the proportion of fat droplet vacuoles, indicating improvement in steatosis. Additionally, there was some improvement in inflammatory cell infiltration in these groups. Similarly, NASH score results show that PPC and HDP reduced elevated NAS to varying degrees in NASH rats (Figure 1C,D). Based on Oil Red O staining results, livers of rats in model group exhibited extremely severe steatosis, with large areas occupied by massive orange‐red fat droplets. Both PPC and HDP effectively reduced the accumulation these fat droplets (Figure 1E,F). These liver pathology findings suggest that PPC and HDP are effective in reducing hepatic pathological changes. Evaluation of liver‐related biochemical parameters indicated that with respect to lipid metabolism, considerable higher levels of TC and TG were present in livers of model group compared to control group (Figure 1G,H). With respect to liver function, model group showed considerable higher serum ALT and AST activities compared to control group (Figure 1I,J). Following PPC or HDP treatment, all of these indicators were improved to different degrees. We also found no significant difference between H‐HDP and PPC groups, suggesting a significant therapeutic effect of HDP on NASH.

### 3.2 Effect of HDP on oxidative stress and inflammatory factors in NASH rat livers

**FIGURE 2:**
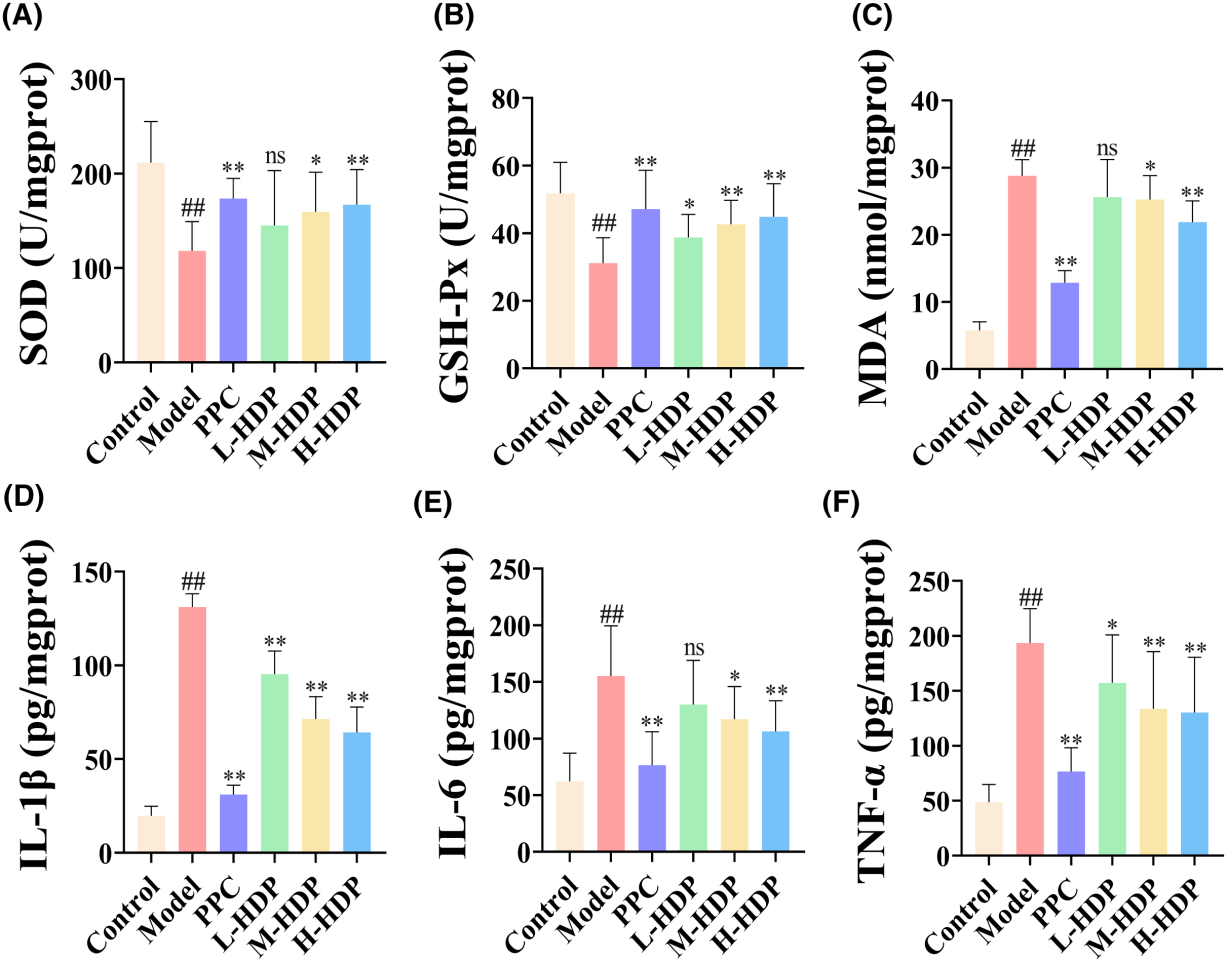
Anti‐oxidative and anti‐inflammatory effects of HDP on NASH rat. HDP significantly improved hepatic oxidative stress and inflammatory factor levels. (A–C) HDP treatment improved the activities of SOD and GSH‐Px, while reducing the level of MDA. (D–F) HDP treatment reduced the levels of hepatic inflammatory cytokines. Data are shown as means ± SD (Control, Model, IRB, L‐HDP, M‐HDP, and H‐HDP groups, *n* = 10 per group). ^##^: *p* < 0.01 versus control group; *: *p* < 0.05 and **: *p* < 0.01 versus model group.

Among the indicators of oxidative stress in livers of NASH rats, activities of SOD and GSH‐Px were decreased compared to control group, while MDA level was increased. (Figure 2A–C). The levels of IL‐1β, IL‐6 and TNF‐α, which are inflammatory factors, were notably increased in livers of model group compared to control group, indicating substantially elevated inflammatory activity in liver (Figure 2D–F). These indicators were reversed in PPC and HDP groups. In the latter, indicators were reversed to varying degrees in a dose‐dependent manner, suggesting that HDP can mitigate elevated oxidative stress and inflammatory factor levels in NASH livers. As no significant difference was found between H‐HDP and PPC groups in treating NASH, we selected H‐HDP group for subsequent mechanistic studies.

### 3.3 Effects of HDP on hepatic metabolites in NASH rats

**FIGURE 3:**
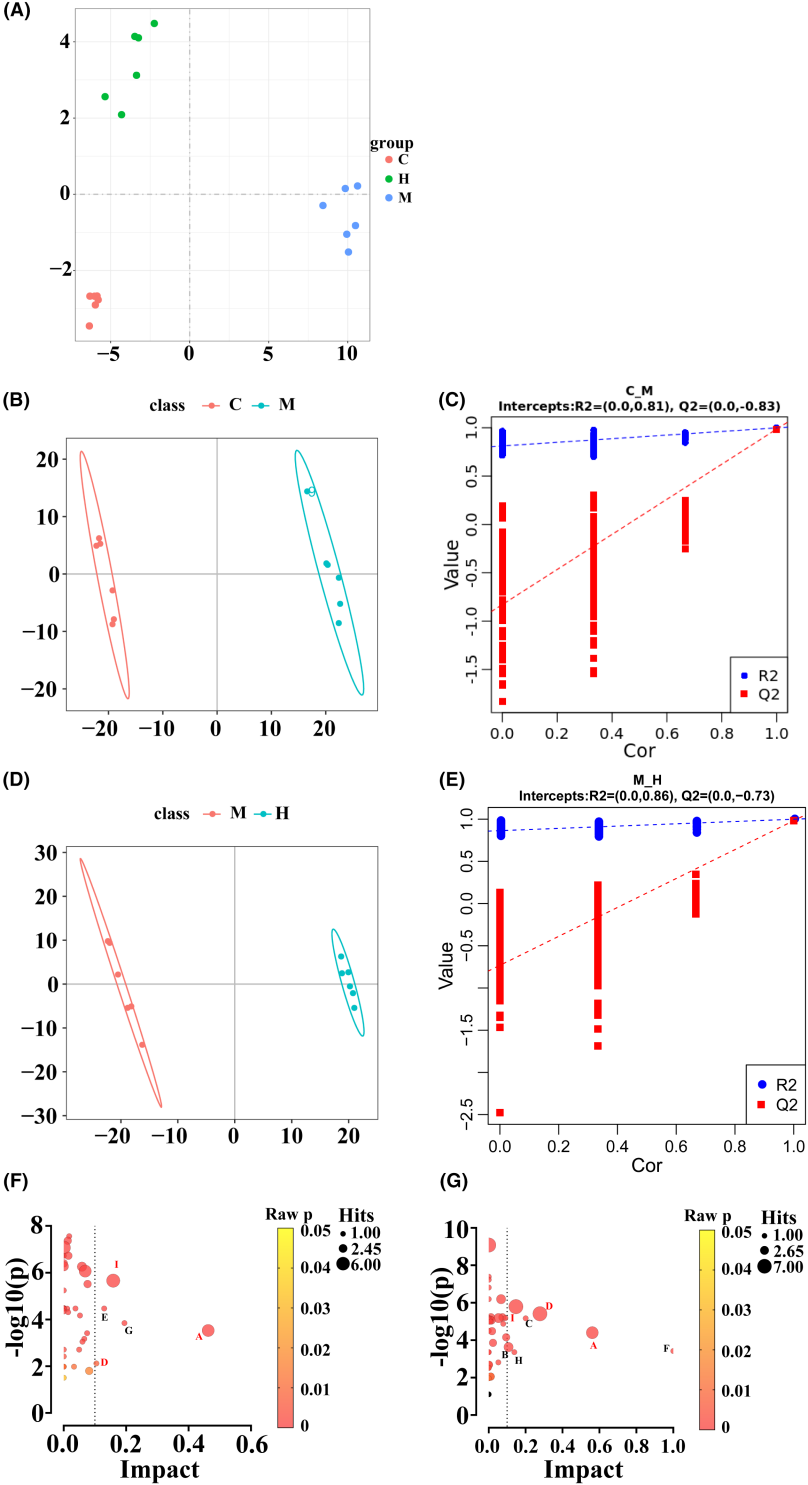
HDP altered hepatic metabolites in NASH rat. The effects of HDP on hepatic metabolites were analysed. (A) Score plots of PCA of control, model, and HDP groups. (B–E) PLS‐DA score plots and permutation tests of the comparison of control versus model, and that of model versus HDP. (F–G) Pathway analysis results of the comparison of control versus model, and that of model versus HDP. Control, Model, and HDP groups, *n* = 6 per group. Name of pathways (A): arachidonic acid metabolism, (B): arginine and proline metabolism, (C): biotin metabolism, (D): glycine‐serine–threonine metabolism, (E): inositol phosphate metabolism, (F): linoleic acid metabolism, (G): nicotinate and nicotinamide metabolism, (H): starch and sucrose metabolism, (I): steroid hormone biosynthesis. Common pathways are marked in red. Black bubbles indicate pathways with *p* ≥ 0.05.

In global principal component analysis (PCA) score plot, control, model, and H‐HDP groups each formed distinct clusters and could be clearly distinguished from each other, indicating that HDP was able to significantly alter NASH‐related liver metabolite abnormalities in rats (Figure 3A). Partial least squares‐discriminant analysis (PLS‐DA) was used to construct prediction model. The PLS‐DA score plots show that the model of control versus model, and that of model versus HDP were significantly separated, with all *R* ^2^Y values above 0.9 and all *Q* ^2^ values below zero, indicating that the model was not subject to overfitting and the results had high explanatory power (Figure 3B–E).

The criteria used to screen for differential metabolites were fold change >1.2, VIP >1.0, and *p* < 0.05. MetaboAnalyst was used for KEGG pathway analysis of differential metabolites. Metabolic pathways that were significantly altered were screened using criteria of *p* < 0.05 and impact >0.1. The results showed that significantly altered metabolic pathways in control versus model included arachidonic acid (AA) metabolism, inositol phosphate metabolism, nicotinate and nicotinamide metabolism, glycine‐serine‐threonine metabolism, and steroid hormone biosynthesis. Those that significantly altered following HDP treatment were AA metabolism, arginine and proline metabolism, linoleic acid metabolism, glycine‐serine‐threonine metabolism, biotin metabolism, starch and sucrose metabolism, and steroid hormone biosynthesis. Among these, AA metabolism, glycine‐serine‐threonine metabolism, steroid hormone biosynthesis are the common metabolic pathways among all three groups. Common differential metabolites were screened between control and model groups and between model and HDP groups (Figure 3F,G). Together with metabolites associated with significantly altered metabolic pathways, a total of 233 differential metabolites were derived, the detailed information was included in supplementary materials (Table S3). Of these, 15 differential metabolites were associated with metabolic pathways that were significantly altered (Table 1).

**TABLE 1:** Differential metabolites related to significantly changed pathways in liver after HDP treatment.

No	Formula	RT [min]	*m*/*z*	Metabolites	VIP		FC		Trend		Pathway
					M versus C	H versus M	M versus C	H versus M	M versus C	H versus M	
1	C_20_H_32_O_2_	9.80	303.23	Arachidonic acid	1.47	1.15	7.56	0.22	↑**	↓^****^	A
2	C_20_H_32_O_3_	8.07	303.23	(±)11(12)‐EET	1.05	1.21	0.36	3.13	↓**	↑^****^	A
3	C_7_H_19_N_3_	1.11	129.14	Spermidine	1.29	1.30	119.28	0.01	↑*	↓****	B
4	C_6_H_14_N_4_O_2_	9.41	175.12	L‐arginine	1.02	1.04	0.55	1.74	↓**	↑	B
5	C_5_H_9_NO_3_	1.32	132.07	Hydroxyproline	1.05	1.02	0.57	1.74	↓**	↑****	B
6	C_4_H_9_N_3_O_2_	1.36	132.08	Creatine	1.27	1.19	22.35	0.06	↑**	↓****	B
7	C_10_H_16_N_2_O_3_S	5.36	245.10	Biotin	1.02	1.14	0.48	2.32	↓**	↑****	C
8	C_4_H_9_NO_3_	1.32	120.07	Threonine	1.51	1.17	0.16	3.90	↓**	↑****	D
9	C_3_H_7_NO_3_	1.33	106.05	Serine	1.19	1.53	0.19	7.29	↓**	↑****	D
10	C_6_H_12_O_6_	1.47	215.03	Inositol	1.15	1.19	0.12	6.27	↓	↑****	E
11	C_18_H_32_O_2_	7.77	279.23	Linoleic Acid	1.44	1.32	0.18	4.44	↓	↑****	F
12	C_6_H_6_N_2_O	1.86	123.06	Nicotinamide	1.25	1.04	2.56	0.47	↑**	↓***	G
13	C_6_H_13_O_9_P	1.47	261.04	d‐Glucose 6‐phosphate	1.29	1.09	0.37	2.16	↓**	↑****	H
14	C_21_H_34_O_4_	6.25	368.28	Tetrahydrocorticosterone	1.42	1.05	3.78	0.38	↑**	↓****	I
15	C_21_H_30_O_4_	6.25	347.22	Corticosterone	1.39	1.31	5.04	0.23	↑**	↓****	I

*Note*: Name of pathways A, arachidonic acid metabolism; B: arginine and proline metabolism; C, biotin metabolism; D, glycine‐serine–threonine metabolism; E, inositol phosphate metabolism; F, linoleic acid metabolism; G, nicotinate and nicotinamide metabolism; H, starch and sucrose metabolism; I, steroid hormone biosynthesis.

Abbreviations: Control group (C), Model group (M), HDP group (H), *n* = 6 per group.

* *p* < 0.05versus control group.

** *p* < 0.01 versus control group.

*** *p* < 0.05 versus control group.

^****^ *p* < 0.01 versus model group.

### 3.4 Effect of HDP on NF‐κB pathway in NASH rats

**FIGURE 4:**
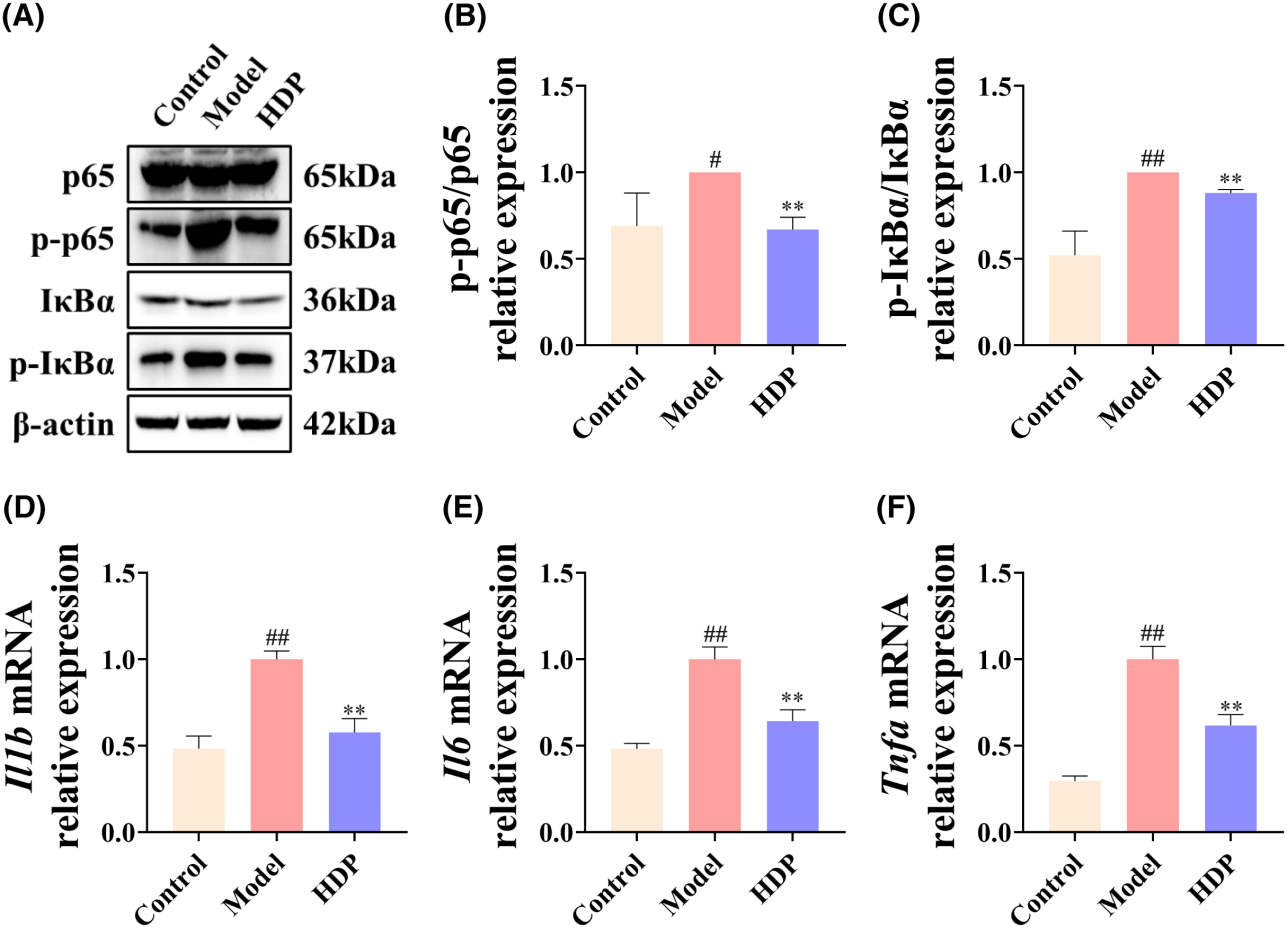
HDP moderated NF‐κB pathway in liver of NASH rat. The expression levels of proteins related to NF‐κB pathway were examined by western blot analysis and the corresponding mRNA levels were examined by RT‐qPCR. (A–C) Changes in phosphorylation levels of p65 and IκBα. (D–F) Relative expression of *Il1b*, *Il6* and *Tnfa* in liver of each group. Data are shown as means ± SD (Control, Model, and HDP groups, *n* = 3 per group). ^##^: *p* < 0.01 versus control group; *: *p* < 0.05 and **: *p* < 0.01 versus model group.

Notably, AA metabolite EET has been shown to inhibit NF‐κB‐mediated inflammatory responses,^16^, ^17^ whereas our results showed that HDP upregulates EET levels. Therefore, we next examined the phosphorylation levels of NF‐κB pathway‐associated proteins p65 and IκBα in liver of control, model, and H‐HDP rats by western blot, and mRNA expression of the cytokines *Il1b*, *Il6* and *Tnfa* by RT‐qPCR. The results showed phosphorylation levels of p65 and IκBα and mRNA levels of *Il1b*, *Il6*, and *Tnfa* were increased in liver of NASH rats. In contrast, all of these indicators decreased to varying degrees following HDP treatment (Figure 4).

### 3.5 Effect of HDP on lipid metabolism‐related pathways in NASH rats

**FIGURE 5:**
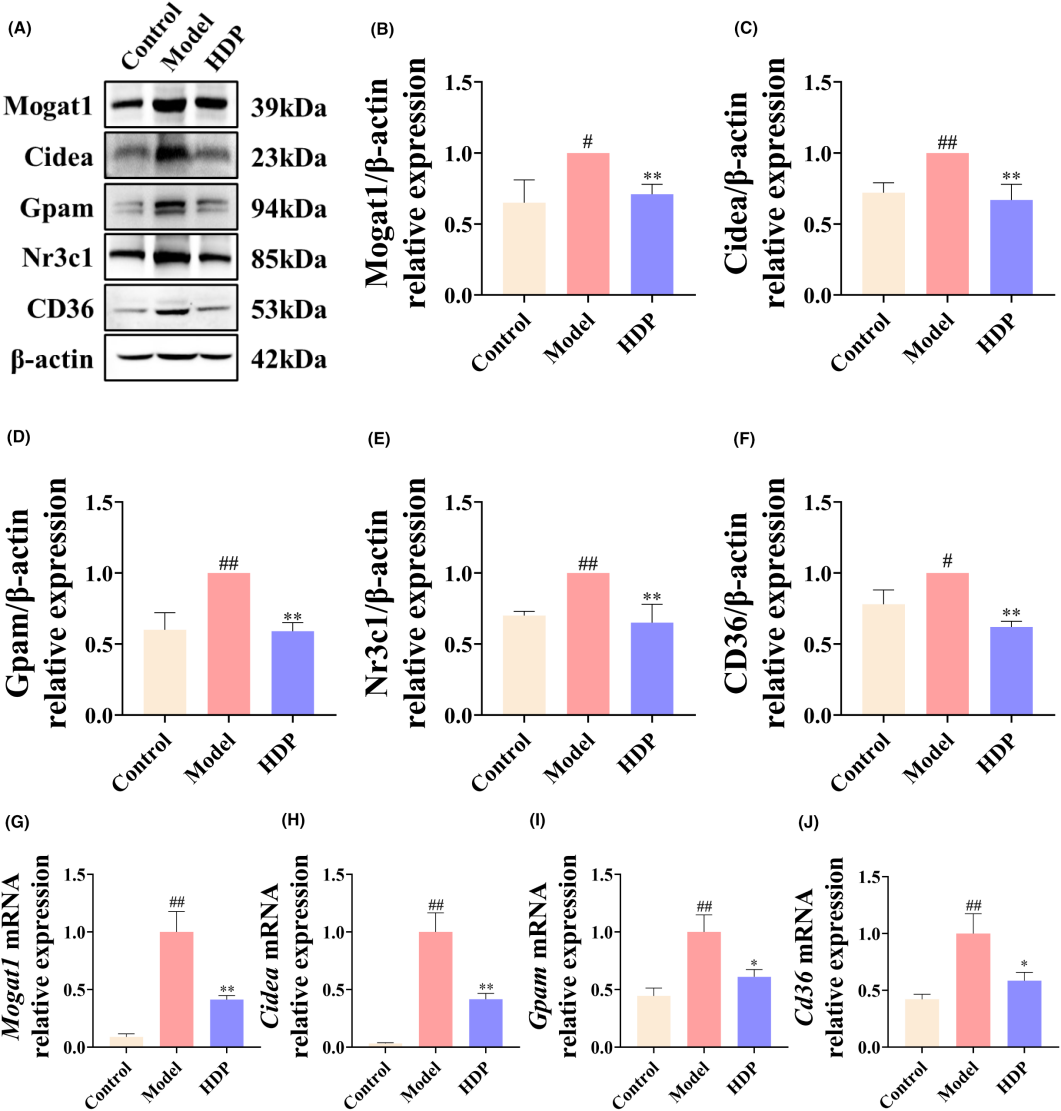
HDP moderated lipid metabolism‐related pathways in liver of NASH rat. The expression levels of proteins related to lipid metabolism‐related pathways were examined by western blot analysis and the corresponding mRNA levels were examined by RT‐qPCR. (A–F) Changes in levels of lipid metabolism‐related proteins Mogat1, Cidea, Gpam, Nr3c1, and CD36. (D–F) Relative expression of *Mogat1*, *Cidea*, *Gpam* and *Cd36* in liver of each group. Data are shown as means ± SD (Control, Model, and HDP groups, *n* = 3 per group). ^##^: *p* < 0.01 versus control group; *: *p* < 0.05 and **: *p* < 0.01 versus model group.

Recent studies have revealed that glycine‐serine‐threonine metabolism, along with steroid hormone biosynthesis, plays a significant role in regulating lipid metabolism.^18^, ^19^, ^20^ We therefore subsequently investigated effects of HDP on lipid metabolism. First, we used RT‐qPCR and western blotting to determine effects of HDP on mRNA and protein expression of genes associated with GR/CD36 pathway of fatty acid uptake. The results showed that levels of GR and CD36 in liver tissues of model group were increased, and that HDP intervention downregulated GR and CD36 expression in liver tissues of NASH rats. In addition, we examined the effects of HDP on mRNA and protein expressions of the Mogat1, Cidea, and Gpam related to lipid metabolism. The results show that Mogat1, Cidea, and Gpam were upregulated in liver of NASH mice, and that HDP intervention downregulated Mogat1, Cidea, and Gpam in liver tissues (Figure 5).

## 4 DISCUSSION

An MCD diet‐induced NASH model was used in present study. Deficiency of methionine and choline prevents the synthesis of very‐low‐density lipoproteins, leading to accumulation of fat in hepatocytes, inflammation, and eventually NASH.^10^ Compared to high‐fat diet (HFD)‐induced NASH models, MCD‐induced models more directly mimic the pathological changes in livers of human NASH patients and therefore remain among the most widely used variants.^11^, ^12^ Results of present study demonstrated that the NASH rats had elevated liver index, significantly elevated serum ALT and AST activities, and elevated hepatic TC and TG levels. HE and Oil Red O staining showed that NASH rats developed considerable fat accumulation in liver, accompanied by a substantial infiltration of inflammatory cells. These results are consistent with those of previous studies.^5^, ^21^ All of these symptoms were considerably improved after HDP or PPC treatment. PPC is frequently used in clinical NASH treatment due to its ability to improve hepatic steatosis by inducing metabolic reprogramming and suppressing inflammation.^22^ H‐HDP and PPC groups did not exhibit significant differences in therapeutic efficacy. This suggests that H‐HDP has excellent potential for treatment of NASH.

Oxidative stress is an important pathophysiological mechanism in NASH. Excessive fat accumulation promotes the production of lipotoxic metabolites such as MDA and the development of oxidative stress in liver.^23^ This in turn leads to depletion of antioxidants such as SOD and GSH‐Px in liver, consistent with clinical observations.^24^ Inflammation plays an equally important role in development of NASH. Under toxic conditions of hepatic steatosis, Kupffer cells and hepatocytes in liver are activated and release excessive inflammatory factors.^25^, ^26^ Accumulated fatty acids can promote maturation of IL‐1β through NLRP3 and maintaining the inflammatory state.^27^ In addition, accumulated fatty acids can also induce lysosomal translocation of Bax, leading to activation of NF‐κB pathway and release of IL‐6 and TNF‐α.^28^ These inflammatory factors are believed to constitute the principal mediators driving development and progression of NASH, as well as onset of associated inflammatory, apoptotic, and fibrotic responses.^29^ In the present study, both HDP and PPC elevated activities of antioxidants SOD and GSH‐Px in liver and decreased levels of lipid peroxidation product MDA and inflammatory cytokines IL‐1β, IL‐6, and TNF‐α. Similarly, H‐HDP and PPC treatments exhibited no significant differences in therapeutic efficacy. Taken together, these results show that H‐HDP has excellent antioxidant and anti‐inflammatory abilities.

11(12)‐EET is a downstream metabolite in AA metabolism. Elevated levels of AA are often detected in patients with NAFLD,^30^ and animal experiments have found that AA is associated with early stages of inflammation and is an early marker of NASH.^31^ EETs have been found to exert anti‐inflammatory effects through PPARγ pathway.^32^, ^33^ Nevertheless, the action of EETs is quickly countered by epoxide hydrolases (sEH, *Ephx2*), which convert them into corresponding dihydroxyeicosatrienoic acids (DHETs), and thus their biological activity tends to be low.^34^ It has recently been shown that promotion of EETs has protective effects against NASH. Mice with targeted knockout or pharmacological inhibition of *Ephx2* exhibited restoration of EET levels in the liver and circulation, as well as a considerable attenuation of liver inflammation and injury^16^, ^17^; this therapeutic effect was also validated in vitro.^35^ Similarly, supplementation with EET analogs reduces adipose tissue expansion, decreases expression of pro‐adipogenic genes, and alleviates glucose intolerance.^36^ Additionally, even direct supplementation with EETs significantly alleviated NASH and downregulated NF‐κB pathway activity in macrophages.^37^ These studies demonstrate that modulation of EET is a promising therapeutic strategy. Results of present study indicated AA content in liver tissue of NASH rats was increased, whereas the 11(12)‐EET content was decreased; both improved following HDP treatment, suggesting that the efficacy of HDP may be associated with regulation of the AA metabolism.

In glycine‐serine‐threonine metabolism, serine and threonine can be interconverted via glycine. Both serine and threonine are important core substrates in the pathway and are involved in numerous biological activities. Serine deficiency has been found to be common in patients with NASH.^38^ Mitochondrial dysfunction in liver is known to inhibit enzymatic reactions involved in the conversion of glycine to serine, resulting in reduced endogenous synthesis of serine.^39^ Supplementation with exogenous serine significantly downregulated TG accumulation and downregulated genes involved in TG synthesis and lipid storage such as *Mogat1*, *Cidea*, *Cidec*, and *Gpam in palmitate‐treated hepatocytes. This effect was shown to be inhibited by knocking out the serine transporters ASCT1 and/or ASCT2*.^20^ Moreover, threonine supplementation significantly ameliorated the symptoms of obese mice and reduced expression levels of adipogenesis‐related genes in epididymal fat, while also upregulating genes associated with lipolysis. In addition, it stimulated the expression of Ucp1 and related genes in brown adipose tissue.^40^ These studies suggest that restoring serine levels may be a promising therapeutic strategy. Present study showed hepatic threonine and serine levels of NASH rats were decreased, whereas these levels increased significantly following HDP treatment. This suggests that the efficacy of HDP may be associated with regulation of serine and threonine.

In steroid hormone biosynthesis, corticosterone (Cort) is a substrate for tetrahydrocorticosterone (THCC). Cort promotes CD36 transcription by binding to glucocorticoid receptor (GR) and thus directly to CD36 promoter.^41^ In contrast, upregulation of CD36 in hepatocytes promotes lipid accumulation in cells, thereby exacerbating NASH.^19^, ^41^ THCC was found to inhibit *Cyp27a1* mRNA expression in goose liver cells,^42^ and CYP27A1 can inhibit steatosis.^43^ Results of present study showed Cort and THCC levels in liver of NASH rats were increased, whereas both were significantly decreased following HDP treatment, suggesting that the efficacy of HDP may be associated with cortisol regulation.

We further validated the effect of HDP on pathways associated with inflammation and lipid metabolism. NF‐κB has been found to play a crucial role in NASH‐associated inflammation.^44^ The main mechanism of NF‐κB activation involves the targeted phosphorylation of IκBα by the multi‐subunit IκB kinase (IKK) complex, leading to the inducible degradation of IκBα, which leads to rapid and transient nuclear translocation of the p65/p50 heterodimer, a canonical member of NF‐κB family.^45^ Infection of MCD‐fed mice with adenovirus expressing nondegradable IκB greatly reduced liver injury and abolished the recruitment of inflammatory cells to liver.^46^ In lipid metabolism, Mogat1 catalyses the synthesis of diacylglycerols, which are precursors of crucial lipids like triacylglycerol (TAG) and phospholipid and widely distributed in stomach, kidney, white and brown adipose tissue, and liver.^47^ Knockdown of *Mogat1* in liver leads to significant reduction in hepatic steatosis in both HFD‐fed mice and ob/ob mice. This effect is accompanied by weight loss and improved glucose tolerance.^48^ Cidea controls the size of lipid droplets,^49^ and depletion of Cidea significantly increases the rate of lipolysis in human adipocytes.^50^ GPAT enzymes are vital in synthesis of hepatic glycerolipids. They facilitate the conversion of glycerol‐3‐phosphate and long‐chain acyl‐CoA into lysophosphatidic acid,^51^ GPAM is a member of GPAT family that is localized on outer mitochondrial membrane and that initiates TAG synthesis and affects phospholipid synthesis.^52^ Rats infected with adenovirus overexpressing *Gpam* exhibited significantly elevated TAG concentrations in liver and plasma.^53^ Conversely, knockdown of hepatic *Gpam* resulted in lower TAG levels in liver and plasma and reduced VLDL‐TAG secretion, which may be associated with a shift in fatty acids from TAG synthesis to oxidation and ketogenesis due to enzyme deficiency.^54^, ^55^ CD36 is known to increase uptake of free fatty acids. In liver, this drives development of cirrhosis and contributes to its progression to NASH.^56^ In recent years, CD36 was also found to be involved in endogenous TG storage, VLDL secretion, and autophagy.^57^, ^58^, ^59^ Knockdown of hepatic *Cd36* prevents systemic inflammation and improves insulin resistance of HFD‐fed mice.^60^ The relationship between CD36 and lipotoxicity remains incompletely understood and requires further investigation. Results of present study showed phosphorylation levels of p65 and IκB and mRNA levels of *Il1b*, *Il6*, and *Tnfa* were increased in liver of NASH rats, suggesting activation of NF‐κB pathway. The hepatic levels of lipid metabolism‐associated proteins Mogat1, Cidea, Gpam, Nr3c1, and CD36 were significantly increased, and the mRNA levels of *Mogat1*, *Cidea*, *Gpam*, and *Cd36* were also elevated, suggesting that fat intake, synthesis, and storage were significantly activated in liver. Following HDP treatment, these inflammation and lipid metabolism‐related changes were corrcted to different degrees, demonstrating that HDP possesses substantial regulatory functionality in both inflammation and lipid metabolism‐related pathways.

## 5 CONCLUSION AND FUTURE PROSPECT

**FIGURE 6:**
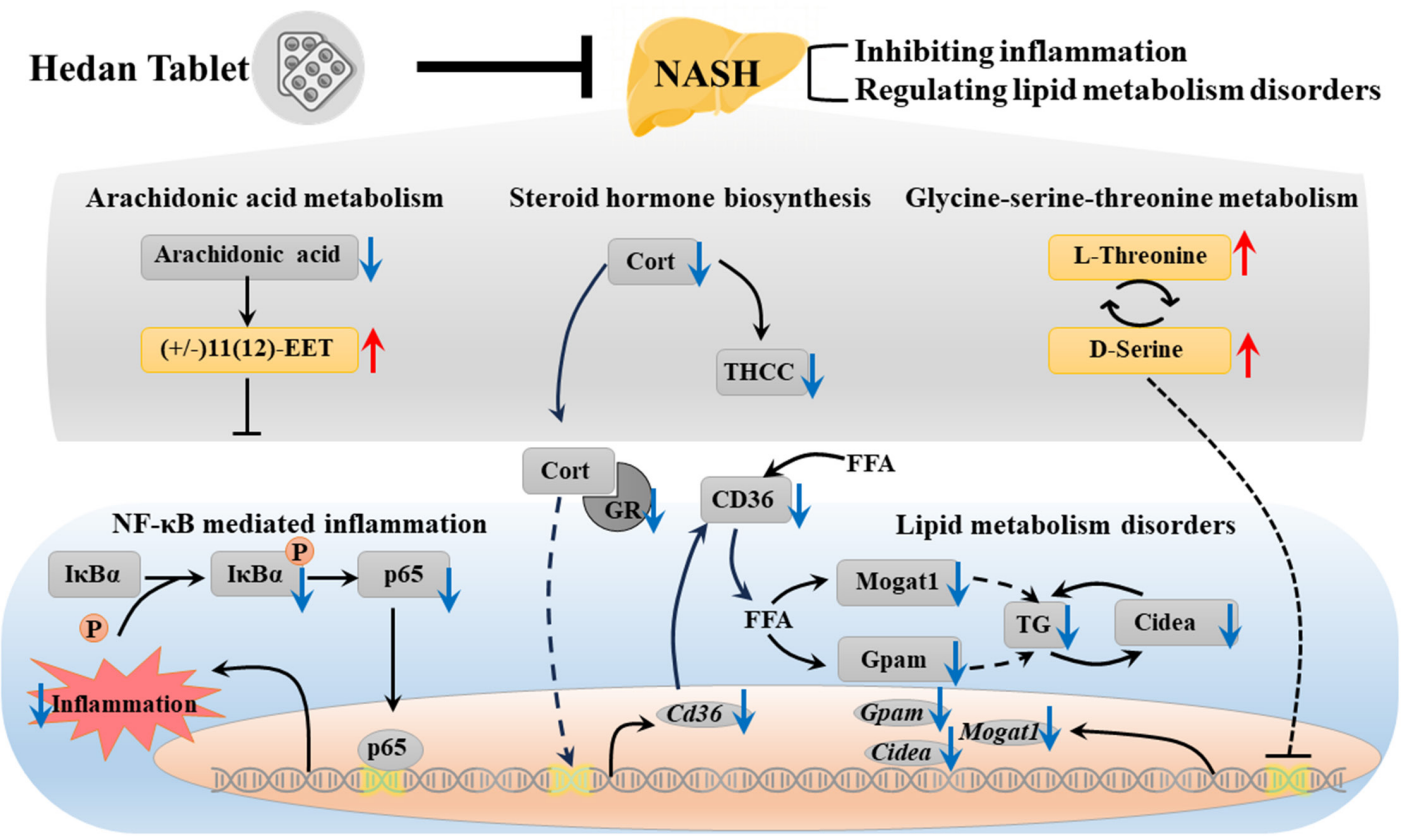
The therapeutic efficacy of HDP in NASH mainly associated with regulation of NF‐κB and lipid metabolism‐related pathways via arachidonic acid metabolism, glycine‐serine‐threonine metabolism, as well as steroid hormone biosynthesis.

In conclusion, the present study found HDP had remarkable therapeutic effects in NASH rats and improved hepatic oxidative stress and inflammatory. The therapeutic efficacy of HDP in NASH may be associated with regulation of NF‐κB and lipid metabolism‐related pathways via AA metabolism, *glycine‐serine*‐*threonine* metabolism, as well as steroid hormone biosynthesis (Figure 6).

There are also some limitations in our study. Compared with HFD‐induced models, MCD‐induced models do not exhibit the characteristics of obesity and insulin resistance exhibited by NASH patients.^61^ However, MCD‐induced models more directly mimic the pathological changes in livers of human NASH patients, including hepatic stestosis, inflammation, oxidative stress, and fibrogenesis. Therefore, MCD‐induced models remain among the most widely used variants.^11^, ^12^ Furthermore, many studies have shown that nuciferine plays a significant role in alleviating hepatic steatosis and mitigating liver injury in HFD‐induced models.^62^, ^63^, ^64^, ^65^ Since nuciferine is the major active component of HDP, we believe that HDP can also exert good therapeutic effects in HFD‐mediated model. Of course, we will verify it and resolve the mechanism in future studies. Besides, studies have shown that remnant cholesterol may play a key role in the development of NAFLD/NASH,^66^, ^67^ while TM6SF2 may play an inhibitory role in this process, and TM6SF2 deficiency promoted the development of hepatic steatosis, hepatic fibrosis and liver cancer.^68^, ^69^ Therefore, in the future study, we will further explore the effect of HDP on remnant cholesterol and TM6SF2 to further understand the mechanism of HDP in NASH treatment.

## AUTHOR CONTRIBUTIONS

**Liying Guo:** Investigation (equal); writing – original draft (equal). **Jinyan Lei:** Investigation (equal); writing – original draft (equal). **Peng Li:** Investigation (equal). **Yuming Wang:** Investigation (equal). **Jing Wang:** Data curation (equal); visualization (equal). **Taotao Song:** Data curation (equal); visualization (equal). **Bo Zhu:** Data curation (equal); visualization (equal). **Jianwei Jia:** Methodology (equal); resources (equal). **Jing Miao:** Conceptualization (equal); funding acquisition (equal); writing – review and editing (equal). **Huantian Cui:** Supervision (equal).

## FUNDING INFORMATION

This research was supported by National Natural Science Foundation of China (82274424) and Tianjin Key Medical Discipline (Specialty) Construction Project (TJYXZDXK‐059B).

## CONFLICT OF INTEREST STATEMENT

The authors declared no conflict of interest.

## Supporting information


Data S1:

## Data Availability

The data that support the findings of this study are available from the corresponding author upon reasonable request.
