# Supplementary material for: Hedan tablet ameliorated non‐alcoholic steatohepatitis by moderating NF‐κB and lipid metabolism‐related pathways via regulating hepatic metabolites
Source: J Cell Mol Med. 2024 Mar 20;28(7):e18194. doi: 10.1111/jcmm.18194 (PMC11967700; doi:10.1111/jcmm.18194)
Supplement: Supplementary file 1 — Data S1: [file JCMM-28-e18194-s001.docx]

**Material and reagents**

MCD chow (45.53% sucrose, 0.75% sodium bicarbonate, 15% corn starch, 5% maltodextrin, 3% cellulose, 10% corn oil, 3.5% S10001 mineral mix, 1% multivitamin V10001, 0% methionine, 0% choline, 0.35% alanine, 1.21% arginine, 0.6% asparagine, 0.35% aspartic acid, 0.35% cystine, 0.4% glutamic acid, 2.33% glycine, 0.45% histidine, 0.82% isoleucine, 1.11% leucine, 1.8% lysine, 0.75% phenylalanine, 0.35% proline, 0.35% serine, 0.82% threonine, 0.18% tryptophan, 0.5% tyrosine, and 0.82% valine) was purchased from Sibeifu Bioscience Co., Ltd. (Beijing, China). Polyene phosphatidylcholine (PPC) was purchased from Sanofi. (Beijing, China). Primary antibody for Mogat1 (AV50240) was purchased from Sigma-Aldrich (Shanghai, China). Primary antibody for Cidea (PA5-115020) was purchased from Thermo Fisher (Shanghai, China). Primary antibodies for p65(bs-20159R), p-p65(bs-0982R),IκBα (bs-1287R),p- IκBα (bsm-52169R), Gpam (bs-5063R), Nr3c1 (bs-13385R), and CD36 (bs-8873R) were purchased from Bioss (Beijing, China). Goat Anti-Rabbit IgG H&L (bs-0295G) and Anti-Mouse IgG H&L antibodies (bs-0296G) were purchased from Bioss (Beijing, China).

**Table S1 Primer sequences**

| Gene name | Organism | Primer sequence (5’-3’) | |
| --- | --- | --- | --- |
| *Actb* | *Rattus norvegicus* | Forward | GGTGGGAATGGGTCAGAAGG |
|  |  | Reverse | TGGCCTTAGGGTTCAGGGG |
| *Il1b* | *Rattus norvegicus* | Forward | AATGCCACCTTTTGACAGTGA |
|  |  | Reverse | GCAGCCCTTCATCTTTTGGG |
| *Il6* | *Rattus norvegicus* | Forward | CCCCAATTTCCAATGCTCTCC |
|  |  | Reverse | GGATGGTCTTGGTCCTTAGCC |
| *Tnf* | *Rattus norvegicus* | Forward | ATGGCCTCCCTCTCATCAGT |
|  |  | Reverse | TTTGCTACGACGTGGGCTAC |
| *Mogat1* | *Rattus norvegicus* | Forward | TCTACACTGTTGCTGCGGTT |
|  |  | Reverse | CTGCCTATCTCGGTCACACC |
| *Cidea* | *Rattus norvegicus* | Forward | GGAGACCGCCAGGGACTA |
|  |  | Reverse | TGAAACCCGGAAAGGACGAG |
| *Gpam* | *Rattus norvegicus* | Forward | AGCTCGGGATTTTGACCTGG |
|  |  | Reverse | TTCAAAGACGGACGGGACAG |
| *Cd36* | *Rattus norvegicus* | Forward | TATTGGTGCTGTCCTGGCTG |
|  |  | Reverse | TGTCTGTACACAGTGGTGCC |

**Metabolomics of liver tissues**

Chromatographic conditions: Chromatographic column: Hypersil Gold column (C18); column temperature: 40℃; flow rate: 0.2 mL/min; positive mode: mobile phase A: 0.1% formic acid; mobile phase B: methanol; negative mode: mobile phase A: 5 mM ammonium acetate and pH 9.0; mobile phase B: methanol. The gradient elution program used is shown in **Table S2**.

**Table S2 The gradient elution program**

| Time (min) | A% | B% |
| --- | --- | --- |
| 0 | 98 | 2 |
| 1.5 | 98 | 2 |
| 3 | 15 | 85 |
| 10 | 0 | 100 |
| 10.1 | 98 | 2 |
| 11 | 98 | 2 |
| 12 | 98 | 2 |

Mass spectrometry conditions: For the purposes of spectrometric analysis, we used a scan range of m/z 100–1500. The ESI source settings were as follows: spray voltage: 3.5 kV; sheath gas flow rate: 35 psi; aux gas flow rate: 10 L/min; capillary temperature: 320℃; lens RF level: 60; aux gas heater temp: 350℃; polarity: positive, negative; MS/MS secondary scans were data dependent.

Data preprocessing and metabolite identification: For processing, the raw data file was imported into CD 3.1 compound discoverer software. For each analyzed metabolite, we performed simple screening based on retention time, mass-to-charge ratio, and other parameters. To align the peaks of different samples to enhance the accuracy of identification, the deviation of retention time was set to 0.2 min and the deviation of mass was set to 5 ppm. The peaks were then extracted by setting the mass deviation to 5 ppm, the signal intensity deviation to 30%, and the signal-to-noise ratio to 3, as well as setting the minimum signal intensity and the addition ion. Having quantified the peak area, the target ion was integrated, and the molecular formula was predicted based on the detected molecular ion peak and fragment ion patterns, which were compared with those of reference spectra in the mzCloud (https://www.mzcloud.org/), mzVault, and the Masslist databases. Blank samples were used to remove the background ions, and the raw quantitative results were normalized to facilitate identification and relative quantification results of the analyzed metabolites. Data processing was based on the Linux operating system (CentOS version 6.6) and R and Python software.

Statistical analysis: For the purposes of metabolite annotation and identification, we used the Kyoto Encyclopedia of Genes and Genomes (KEGG) (https://www.genome.jp/kegg/pathway.html), Human Metabolome (https://hmdb.ca/metabolites), and LIPID MAPS (http://www.lipidmaps.org/) databases. For multivariate analysis, we used metaX metabolomics data processing software (Wen, Mei, Zeng, & Liu, 2017) for data conversion. The same software was used for principal component analysis (PCA) and partial least squares-discriminant analysis (PLS-DA) to determine the variable importance in projection (VIP) scores of each metabolite. For univariate analysis, the statistical significance (P value) of difference in each metabolite between two groups was determined using *t* tests, and we calculated metabolite fold changes (FC) between the two groups. The default criteria for screening differential metabolites were a VIP >1 and *P* < 0.05, with an FC ≥ 1.2 or FC ≤ 0.83. Finally, the differential metabolites identified via screening were imported into MetaboAnalyst 5.0 for analysis of metabolic pathway enrichment.

**Table S3 Differential metabolites**

| No. | Formula | RT [min] | m/z | Metabolites | VIP | | FC | | Trend | | Pathway |
| --- | --- | --- | --- | --- | --- | --- | --- | --- | --- | --- | --- |
|  |  |  |  |  | M vs. C | H vs. M | M vs. C | H vs. M | M vs. C | H vs. M |  |
| 1 | C26 H43 N O5 | 6.70 | 448.31 | Glycodeoxycholic Acid (hydrate) | 1.53 | 1.03 | 602.73 | 0.02 | ↑## | ↓** |  |
| 2 | C15 H16 O2 | 5.83 | 227.11 | Bisphenol A | 1.48 | 1.29 | 34.17 | 0.05 | ↑## | ↓** |  |
| 3 | C18 H32 O3 | 7.33 | 341.23 | (±)9(10)-EpOME | 1.47 | 1.29 | 8.63 | 0.16 | ↑## | ↓** |  |
| 4 | C17 H28 N6 O4 | 7.27 | 341.23 | KPH | 1.46 | 1.31 | 8.27 | 0.16 | ↑## | ↓** |  |
| 5 | C28 H48 N O7 P | 8.40 | 586.32 | LPC 20:5 | 1.43 | 1.34 | 0.05 | 16.43 | ↓## | ↑** |  |
| 6 | C20 H30 O5 | 7.67 | 331.19 | 19(R)-hydroxy Prostaglandin A2 | 1.34 | 1.43 | 23.61 | 0.04 | ↑## | ↓** |  |
| 7 | C16 H30 O2 | 9.61 | 253.22 | Palmitoleic Acid | 1.48 | 1.28 | 0.09 | 7.45 | ↓## | ↑** |  |
| 8 | C20 H34 O5 | 7.40 | 377.23 | 5-trans prostaglandin F2β | 1.46 | 1.23 | 8.26 | 0.19 | ↑## | ↓** |  |
| 9 | C26 H36 O4 | 7.14 | 413.27 | 5-[(10Z)-14-(3,5-dihydroxyphenyl)tetradec-10-en-1-yl]benzene-1,3-diol | 1.50 | 1.22 | 35.53 | 0.06 | ↑## | ↓** |  |
| 10 | C21 H36 O4 | 8.82 | 351.25 | MAG (18:3) | 1.41 | 1.39 | 29.85 | 0.04 | ↑## | ↓** |  |
| 11 | C15 H22 O3 | 6.85 | 249.15 | 4-(octyloxy)benzoic acid | 1.30 | 1.44 | 0.04 | 35.04 | ↓## | ↑** |  |
| 12 | C12 H18 O2 | 6.95 | 411.25 | Sedanolide | 1.50 | 1.05 | 26.51 | 0.13 | ↑## | ↓** |  |
| 13 | C22 H34 O2 | 10.01 | 329.25 | Docosapentaenoic acid | 1.50 | 1.23 | 0.02 | 19.47 | ↓## | ↑** |  |
| 14 | C11 H11 N O3 | 5.27 | 206.08 | Cinnamoylglycine | 1.33 | 1.47 | 25.55 | 0.03 | ↑## | ↓** |  |
| 15 | C26 H43 N O6 | 6.78 | 464.30 | Glycocholic acid | 1.53 | 1.07 | 308.42 | 0.03 | ↑## | ↓** |  |
| 16 | C22 H32 O4 | 8.05 | 359.22 | 17(S)-HpDHA | 1.47 | 1.27 | 15.59 | 0.10 | ↑## | ↓** |  |
| 17 | C4 H9 N O3 | 1.32 | 120.07 | Threonine | 1.51 | 1.17 | 0.16 | 3.90 | ↓## | ↑** | D |
| 18 | C22 H32 O2 | 9.72 | 327.23 | Docosahexaenoic acid | 1.41 | 1.38 | 7.35 | 0.15 | ↑## | ↓** |  |
| 19 | C24 H38 O4 | 6.99 | 389.27 | 7-Ketolithocholic acid | 1.51 | 1.15 | 31.86 | 0.08 | ↑## | ↓** |  |
| 20 | C20 H36 O4 | 9.23 | 341.27 | 11-Deoxy prostaglandin F1α | 1.39 | 1.31 | 4.52 | 0.26 | ↑## | ↓** |  |
| 21 | C19 H26 O4 | 7.39 | 319.19 | Coenzyme Q2 | 1.53 | 1.07 | 12.31 | 0.18 | ↑## | ↓** |  |
| 22 | C7 H19 N3 | 1.11 | 129.14 | Spermidine | 1.29 | 1.30 | 119.28 | 0.01 | ↑# | ↓* | B |
| 23 | C30 H48 O10 | 7.57 | 569.33 | Chenodeoxycholic acid-3-beta-D-glucuronide | 1.36 | 1.01 | 1.30 | 2.11 | ↑# | ↑* |  |
| 24 | C16 H26 O5 | 6.83 | 279.16 | 13,14-dihydro-15-keto-tetranor Prostaglandin D2 | 1.46 | 1.31 | 17.23 | 0.09 | ↑## | ↓** |  |
| 25 | C11 H20 O2 | 6.99 | 185.15 | 10-Undecenoic acid | 1.49 | 1.20 | 33.46 | 0.06 | ↑## | ↓** |  |
| 26 | C19 H34 O4 | 8.47 | 349.23 | 1,2-dihydroxyheptadec-16-yn-4-yl acetate | 1.45 | 1.23 | 3.52 | 0.36 | ↑## | ↓** |  |
| 27 | C18 H16 Cl N O | 4.81 | 296.08 | 3-anilino-5-(4-chlorophenyl)cyclohex-2-en-1-one | 1.44 | 1.12 | 0.03 | 21.06 | ↓## | ↑* |  |
| 28 | C22 H34 O4 | 8.44 | 385.23 | 16,16-Dimethyl prostaglandin A2 | 1.50 | 1.06 | 9.88 | 0.22 | ↑## | ↓** |  |
| 29 | C17 H32 O2 | 10.04 | 267.23 | trans-10-Heptadecenoic Acid | 1.54 | 1.06 | 0.12 | 3.94 | ↓## | ↑** |  |
| 30 | C28 H52 N O7 P | 9.07 | 546.36 | PC (18:3e/2:0) | 1.24 | 1.50 | 0.27 | 4.62 | ↓## | ↑** |  |
| 31 | C20 H30 O2 | 9.64 | 303.23 | Methyltestosterone | 1.28 | 1.44 | 0.28 | 4.01 | ↓## | ↑** |  |
| 32 | C24 H40 O4 | 7.99 | 393.30 | Ursodeoxycholic acid | 1.40 | 1.29 | 18.83 | 0.08 | ↑## | ↓** |  |
| 33 | C20 H32 O2 | 9.80 | 303.23 | Arachidonic acid | 1.47 | 1.15 | 7.56 | 0.22 | ↑## | ↓** | A |
| 34 | C11 H15 N3 O6 | 4.88 | 286.10 | N4-Acetylcytidine | 1.40 | 1.36 | 0.48 | 1.98 | ↓## | ↑** |  |
| 35 | C34 H58 O4 | 9.61 | 529.42 | FAHFA (16:1/18:3) | 1.52 | 1.10 | 0.03 | 10.64 | ↓## | ↑** |  |
| 36 | C20 H24 O6 | 7.07 | 343.15 | 2,5-bis(4-hydroxy-3-methoxyphenyl)-3,4-dimethyloxolan-3-ol | 1.44 | 1.27 | 4.77 | 0.27 | ↑## | ↓** |  |
| 37 | C25 H50 N O7 P | 9.08 | 570.34 | LPC 17:1 | 1.33 | 1.41 | 0.25 | 4.10 | ↓## | ↑** |  |
| 38 | C16 H30 N4 O6 | 10.00 | 375.23 | DLK | 1.41 | 1.36 | 0.03 | 29.20 | ↓## | ↑** |  |
| 39 | C27 H46 O4 S | 6.06 | 465.31 | cholesteryl sulfate | 1.45 | 1.32 | 159.25 | 0.01 | ↑## | ↓** |  |
| 40 | C18 H28 O3 | 7.68 | 275.20 | 12-Oxo phytodienoic acid | 1.45 | 1.29 | 18.70 | 0.08 | ↑## | ↓** |  |
| 41 | C27 H46 O2 | 10.35 | 401.34 | delta-Tocopherol | 1.27 | 1.46 | 2.45 | 0.37 | ↑## | ↓** |  |
| 42 | C11 H13 N O3 | 5.22 | 225.12 | DL-2-(acetylamino)-3-phenylpropanoic acid | 1.13 | 1.55 | 0.13 | 15.43 | ↓## | ↑** |  |
| 43 | C6 H14 N4 O2 | 9.41 | 175.12 | L-arginine | 1.02 | 1.04 | 0.55 | 1.74 | ↓## | ↑ | B |
| 44 | C20 H32 O3 | 8.07 | 343.22 | 16(R)-HETE | 1.52 | 1.84 | 0.84 | 1.29 | ↓ | ↑* |  |
| 45 | C26 H42 N Na O6 | 6.18 | 532.29 | Glycohyocholic acid Sodium salt | 1.48 | 1.22 | 110.35 | 0.03 | ↑## | ↓** |  |
| 46 | C42 H64 O4 | 9.76 | 631.47 | FAHFA (20:4/22:5) | 1.45 | 1.20 | 0.07 | 8.66 | ↓## | ↑** |  |
| 47 | C18 H25 F N4 O3 | 10.00 | 387.18 | 5-fluoro AB-PINACA N-(4-hydroxypentyl) metabolite | 1.40 | 1.34 | 0.05 | 15.72 | ↓## | ↑** |  |
| 48 | C14 H28 O2 | 9.39 | 227.20 | Myristic acid | 1.45 | 1.28 | 0.26 | 3.16 | ↓## | ↑** |  |
| 49 | C24 H48 N O7 P | 8.64 | 538.32 | LPC 16:1 | 1.17 | 1.54 | 0.33 | 4.06 | ↓## | ↑** |  |
| 50 | C25 H42 N O7 P | 8.37 | 500.28 | LPE 20:5 | 1.22 | 1.47 | 0.18 | 7.61 | ↓## | ↑** |  |
| 51 | C19 H37 O7 P | 8.64 | 407.22 | LPA 16:1 | 1.34 | 1.40 | 0.24 | 4.27 | ↓## | ↑** |  |
| 52 | C27 H40 O4 | 8.75 | 429.30 | Hydroxyprogesterone caproate | 1.29 | 1.47 | 9.70 | 0.08 | ↑## | ↓** |  |
| 53 | C24 H40 O3 | 8.36 | 375.29 | Allolithocholic acid | 1.34 | 1.45 | 4.28 | 0.22 | ↑## | ↓** |  |
| 54 | C21 H36 O3 | 6.31 | 359.26 | Pregnanetriol | 1.45 | 1.22 | 9.14 | 0.17 | ↑## | ↓** |  |
| 55 | C24 H40 O5 | 6.63 | 815.57 | β-Muricholic acid | 1.43 | 1.16 | 2203.05 | 0.00 | ↑## | ↓** |  |
| 56 | C18 H20 N2 O3 | 7.96 | 313.15 | Phe-Phe | 1.35 | 1.43 | 7.86 | 0.12 | ↑## | ↓** |  |
| 57 | C3 H7 N O3 | 1.33 | 106.05 | Serine | 1.19 | 1.53 | 0.19 | 7.29 | ↓## | ↑** | D |
| 58 | C7 H14 N2 O3 | 1.40 | 175.11 | N-Acetylornithine | 1.35 | 1.38 | 3.55 | 0.29 | ↑## | ↓** |  |
| 59 | C30 H52 N O7 P | 9.11 | 614.35 | LPC 22:5 | 1.24 | 1.50 | 0.12 | 11.55 | ↓## | ↑** |  |
| 60 | C5 H9 N O3 | 1.32 | 132.07 | Hydroxyproline | 1.05 | 1.02 | 0.57 | 1.74 | ↓## | ↑** | B |
| 61 | C18 H32 O3 | 7.97 | 295.23 | 12,13-EODE | 1.41 | 1.32 | 0.67 | 3.96 | ↓## | ↑** |  |
| 62 | C27 H54 N O7 P | 9.69 | 536.37 | PC (14:1e/5:0) | 1.39 | 1.35 | 0.16 | 5.42 | ↓## | ↑** |  |
| 63 | C20 H26 O3 | 7.01 | 315.19 | Kahweol | 1.25 | 1.40 | 0.34 | 3.32 | ↓## | ↑** |  |
| 64 | C24 H50 N O7 P | 9.72 | 494.33 | 1-Palmitoyl-Sn-Glycero-3-Phosphocholine | 1.45 | 1.21 | 0.30 | 2.63 | ↓## | ↑** |  |
| 65 | C19 H36 O4 | 9.14 | 351.25 | 2,4-dihydroxyheptadec-16-en-1-yl acetate | 1.32 | 1.42 | 0.09 | 11.02 | ↓## | ↑** |  |
| 66 | C18 H30 O4 | 7.68 | 311.22 | 9-HpOTrE | 1.44 | 1.33 | 28.06 | 0.05 | ↑## | ↓** |  |
| 67 | C20 H36 O6 | 7.48 | 371.24 | 13,14-dihydro-19(R)-hydroxy Prostaglandin E1 | 1.39 | 1.33 | 7.81 | 0.14 | ↑## | ↓** |  |
| 68 | C16 H32 O2 | 10.25 | 255.23 | Palmitic acid | 1.49 | 1.11 | 0.40 | 1.92 | ↓## | ↑** |  |
| 69 | C48 H76 N O7 P | 6.89 | 810.54 | PC (22:6e/18:5) | 1.26 | 1.12 | 0.41 | 2.24 | ↓## | ↑** |  |
| 70 | C18 H32 O2 | 7.77 | 279.23 | Linoleic Acid | 1.44 | 1.32 | 0.18 | 4.44 | ↓ | ↑** | F |
| 71 | C30 H50 N O7 P | 8.81 | 612.33 | LPC 22:6 | 1.09 | 1.31 | 0.96 | 2.33 | ↓ | ↑** |  |
| 72 | C12 H20 O5 | 5.77 | 243.12 | 3,8,9-trihydroxy-10-propyl-3,4,5,8,9,10-hexahydro-2H-oxecin-2-one | 1.43 | 1.20 | 4.56 | 0.31 | ↑## | ↓** |  |
| 73 | C24 H40 O4 | 7.91 | 391.29 | Deoxycholic acid | 1.31 | 1.12 | 26.20 | 0.11 | ↑## | ↓** |  |
| 74 | C48 H86 N O8 P | 7.15 | 836.61 | PC (20:2/20:3) | 1.04 | 1.36 | 13.01 | 0.07 | ↑## | ↓** |  |
| 75 | C20 H30 O5 | 7.56 | 333.20 | Prostaglandin K2 | 1.50 | 1.05 | 4.06 | 0.39 | ↑## | ↓** |  |
| 76 | C21 H32 O5 | 7.94 | 365.23 | Tetrahydroaldosterone | 1.36 | 1.41 | 2.82 | 0.36 | ↑## | ↓** |  |
| 77 | C25 H52 N O7 P | 9.72 | 554.35 | LPC 17:0 | 1.47 | 1.06 | 0.33 | 2.15 | ↓## | ↑** |  |
| 78 | C10 H16 N2 O2 | 5.34 | 197.13 | 3-(propan-2-yl)-octahydropyrrolo[1,2-a]pyrazine-1,4-dione | 1.16 | 1.04 | 0.28 | 3.53 | ↓## | ↑** |  |
| 79 | C6 H13 O9 P | 1.47 | 261.04 | D-Glucose 6-phosphate | 1.29 | 1.09 | 0.37 | 2.16 | ↓## | ↑** | H |
| 80 | C20 H32 O3 | 8.07 | 319.23 | 11,12-Epoxy-(5Z,8Z,11Z)-icosatrienoic acid | 1.77 | 1.46 | 0.42 | 4.76 | ↓## | ↑** |  |
| 81 | C22 H34 O4 | 9.16 | 361.24 | (±)7(8)-DiHDPA | 1.49 | 1.24 | 7.35 | 0.20 | ↑## | ↓** |  |
| 82 | C27 H52 N O7 P | 9.38 | 578.35 | LPC 19:2 | 1.44 | 1.25 | 0.15 | 4.79 | ↓## | ↑** |  |
| 83 | C26 H52 N O7 P | 9.50 | 566.35 | LPC 18:1 | 1.42 | 1.29 | 0.44 | 2.07 | ↓## | ↑** |  |
| 84 | C25 H52 N O7 P | 10.13 | 508.34 | Lysopc 17:0 | 1.26 | 1.39 | 0.41 | 2.58 | ↓## | ↑** |  |
| 85 | C25 H48 F O5 P | 8.29 | 501.32 | O-7460 | 1.53 | 1.05 | 123.10 | 0.05 | ↑## | ↓** |  |
| 86 | C18 H30 O5 | 7.36 | 307.19 | 2,3-dinor Prostaglandin E1 | 1.38 | 1.38 | 17.87 | 0.06 | ↑## | ↓** |  |
| 87 | C21 H44 N O7 P | 9.22 | 452.28 | LPE 16:0 | 1.22 | 1.34 | 0.59 | 1.75 | ↓## | ↑** |  |
| 88 | C24 H40 O4 | 7.92 | 437.29 | Chenodeoxycholic Acid | 1.30 | 1.09 | 24.79 | 0.11 | ↑## | ↓** |  |
| 89 | C4 H9 N O3 | 1.32 | 118.05 | Threonine | 1.26 | 1.44 | 3.37 | 0.26 | ↑## | ↓** |  |
| 90 | C23 H38 O5 | 6.31 | 393.27 | 23-Norcholic acid | 1.42 | 1.10 | 12.21 | 0.17 | ↑## | ↓** |  |
| 91 | C15 H19 N O6 | 5.61 | 308.12 | methyl 5-{[2-(ethoxycarbonyl)-3-oxohex-1-enyl]amino}-2-furoate | 1.27 | 1.05 | 0.16 | 5.31 | ↓## | ↑* |  |
| 92 | C11 H16 N2 O2 | 7.20 | 105.07 | Pilocarpine | 1.39 | 1.16 | 10.83 | 0.16 | ↑## | ↓** |  |
| 93 | C26 H54 N O7 P | 10.54 | 522.36 | LysoPC 18:0 | 1.35 | 1.30 | 0.26 | 3.57 | ↓## | ↑** |  |
| 94 | C23 H48 N O7 P | 9.29 | 480.31 | LysoPE 18:0 | 1.34 | 1.25 | 0.54 | 1.75 | ↓## | ↑** |  |
| 95 | C14 H19 N S | 5.37 | 234.13 | 4-(1-adamantyl)-2-methyl-1,3-thiazole | 1.51 | 1.06 | 11.80 | 0.19 | ↑## | ↓** |  |
| 96 | C9 H9 N O3 | 5.36 | 180.07 | Hippuric acid | 1.19 | 1.50 | 0.26 | 4.95 | ↓## | ↑** |  |
| 97 | C21 H41 O7 P | 9.50 | 435.25 | Oleoyl-L-alpha-lysophosphatidic acid | 1.46 | 1.09 | 0.27 | 2.63 | ↓## | ↑** |  |
| 98 | C11 H14 O5 | 5.77 | 225.08 | Methyl EudesMate | 1.40 | 1.04 | 4.67 | 0.35 | ↑## | ↓** |  |
| 99 | C13 H14 O5 | 5.61 | 233.08 | Citrinin | 1.31 | 1.07 | 2.82 | 0.47 | ↑## | ↓** |  |
| 100 | C11 H17 N5 O5 | 1.32 | 298.11 | N7-Methylguanosine | 1.21 | 1.47 | 2.93 | 0.28 | ↑## | ↓** |  |
| 101 | C21 H40 O4 | 9.92 | 357.30 | Monoolein | 1.26 | 1.39 | 0.14 | 7.93 | ↓## | ↑** |  |
| 102 | C30 H52 N O7 P | 9.11 | 570.35 | PC (18:5e/4:0) | 1.14 | 1.55 | 0.17 | 9.32 | ↓## | ↑** |  |
| 103 | C25 H26 N2 O3 | 6.97 | 403.21 | 2-piperidinophenyl N-[4-(benzyloxy)phenyl]carbamate | 1.23 | 1.35 | 2.61 | 0.37 | ↑## | ↓** |  |
| 104 | C43 H78 N O7 P | 9.32 | 796.55 | PE (18:1e/20:4) | 1.43 | 1.28 | 0.05 | 13.23 | ↓## | ↑** |  |
| 105 | C9 H13 N O3 | 5.03 | 184.10 | L-Adrenaline | 1.24 | 1.44 | 0.34 | 3.35 | ↓## | ↑** |  |
| 106 | C20 H30 O3 | 7.68 | 317.21 | (±)18-HEPE | 1.41 | 1.26 | 0.04 | 13.06 | ↓## | ↑** |  |
| 107 | C14 H18 O4 | 6.14 | 249.11 | 2-(2-carboxy-2-methylpropyl)-4,6-dimethylbenzoic acid | 1.40 | 1.35 | 9.22 | 0.13 | ↑## | ↓** |  |
| 108 | C15 H24 O4 | 6.82 | 267.16 | 3-[4-methyl-1-(2-methylpropanoyl)-3-oxocyclohexyl]butanoic acid | 1.06 | 1.34 | 0.33 | 4.15 | ↓## | ↑** |  |
| 109 | C25 H52 N O7 P | 9.59 | 510.36 | PC (14:0e/3:0) | 1.42 | 1.17 | 0.34 | 2.30 | ↓## | ↑** |  |
| 110 | C21 H34 O4 | 6.25 | 368.28 | Tetrahydrocorticosterone | 1.42 | 1.05 | 3.78 | 0.38 | ↑## | ↓** | I |
| 111 | C14 H20 O2 | 7.11 | 221.15 | 2,6-Di-tert-butyl-1,4-benzoquinone | 1.02 | 1.46 | 2.45 | 0.30 | ↑## | ↓** |  |
| 112 | C28 H56 N O7 P | 10.31 | 550.39 | PC (18:1e/2:0) | 1.34 | 1.24 | 0.62 | 1.53 | ↓## | ↑** |  |
| 113 | C20 H32 N4 O4 S | 7.57 | 447.20 | FMK | 1.33 | 1.40 | 9.58 | 0.10 | ↑## | ↓** |  |
| 114 | C28 H35 N3 O7 | 7.45 | 508.24 | Virginiamycin | 1.34 | 1.43 | 21.49 | 0.04 | ↑## | ↓** |  |
| 115 | C17 H34 N6 O5 | 9.39 | 403.26 | QKK | 1.38 | 1.11 | 0.21 | 3.20 | ↓## | ↑** |  |
| 116 | C14 H28 O3 | 8.19 | 243.20 | (R)-3-Hydroxy myristic acid | 1.43 | 1.06 | 0.42 | 1.84 | ↓## | ↑** |  |
| 117 | C21 H30 O4 | 6.25 | 347.22 | Corticosterone | 1.39 | 1.31 | 5.04 | 0.23 | ↑## | ↓** | I |
| 118 | C15 H28 O2 | 7.66 | 223.21 | 7-(2-hydroxypropan-2-yl)-1,4a-dimethyl-decahydronaphthalen-1-ol | 1.28 | 1.22 | 0.35 | 2.61 | ↓## | ↑** |  |
| 119 | C17 H20 O9 | 5.21 | 367.10 | 3-O-Feruloylquinic acid | 1.09 | 1.04 | 0.38 | 3.00 | ↓## | ↑ |  |
| 120 | C22 H36 O2 | 10.47 | 333.28 | Adrenic acid | 1.12 | 1.14 | 0.34 | 3.16 | ↓## | ↑** |  |
| 121 | C20 H36 O4 | 6.33 | 363.25 | 11-Deoxy prostaglandin F1β | 1.41 | 1.29 | 10.36 | 0.13 | ↑## | ↓** |  |
| 122 | C24 H48 N O7 P | 8.42 | 494.32 | PC (14:1e/2:0) | 1.01 | 1.50 | 0.42 | 3.53 | ↓## | ↑** |  |
| 123 | C28 H48 N O7 P | 8.22 | 542.32 | PC (18:5e/2:0) | 1.35 | 1.35 | 0.06 | 15.62 | ↓## | ↑** |  |
| 124 | C23 H48 N O7 P | 8.84 | 526.32 | LPC 15:0 | 1.25 | 1.39 | 0.54 | 1.90 | ↓## | ↑** |  |
| 125 | C22 H34 O4 | 8.33 | 361.24 | (±)19(20)-DiHDPA | 1.21 | 1.45 | 2.29 | 0.38 | ↑## | ↓** |  |
| 126 | C14 H26 O4 | 6.98 | 257.18 | Tetradecanedioic acid | 1.38 | 1.33 | 10.81 | 0.11 | ↑## | ↓** |  |
| 127 | C15 H24 N6 O6 | 10.01 | 385.18 | TQH | 1.27 | 1.30 | 0.09 | 10.87 | ↓## | ↑** |  |
| 128 | C8 H9 N O2 | 1.21 | 152.07 | Paracetamol | 1.02 | 1.43 | 1.90 | 0.43 | ↑## | ↓** |  |
| 129 | C20 H30 O3 | 7.68 | 301.22 | (±)8-HEPE | 1.42 | 1.25 | 0.05 | 11.17 | ↓## | ↑** |  |
| 130 | C4 H8 O4 | 1.48 | 119.04 | D-Threose | 1.14 | 1.48 | 3.98 | 0.17 | ↑## | ↓** |  |
| 131 | C46 H84 N O8 P | 6.59 | 872.60 | PC (18:0/20:4) | 1.37 | 1.38 | 26.69 | 0.04 | ↑## | ↓** |  |
| 132 | C28 H54 N O7 P | 9.56 | 548.37 | PC (18:2e/2:0) | 1.24 | 1.47 | 0.38 | 2.95 | ↓## | ↑** |  |
| 133 | C28 H52 N O7 P | 9.24 | 590.35 | LPC 20:3 | 1.34 | 1.40 | 0.21 | 4.82 | ↓## | ↑** |  |
| 134 | C4 H6 N4 O3 | 1.35 | 157.04 | Allantoin | 1.22 | 1.35 | 0.50 | 2.10 | ↓## | ↑** |  |
| 135 | C9 H9 N | 5.84 | 130.07 | 3-Methylindole | 1.35 | 1.29 | 8.56 | 0.14 | ↑## | ↓** |  |
| 136 | C18 H34 O2 | 9.50 | 281.25 | Oleic Acid | 1.35 | 1.25 | 0.47 | 1.97 | ↓## | ↑** |  |
| 137 | C5 H8 O4 | 1.31 | 131.04 | Glutaric Acid | 1.43 | 1.22 | 0.40 | 2.11 | ↓## | ↑** |  |
| 138 | C22 H21 Cl N2 O8 | 6.12 | 475.09 | Meclocycline | 1.35 | 1.38 | 3.96 | 0.26 | ↑## | ↓** |  |
| 139 | C21 H40 O4 | 9.72 | 355.29 | Mag (18:1) | 1.15 | 1.43 | 2.90 | 0.28 | ↑## | ↓** |  |
| 140 | C49 H88 O10 | 9.91 | 895.64 | MGDG (22:1/18:2) | 1.08 | 1.45 | 0.41 | 3.19 | ↓## | ↑** |  |
| 141 | C22 H38 O2 | 10.96 | 333.28 | Docosatrienoic Acid | 1.35 | 1.26 | 0.15 | 5.37 | ↓## | ↑** |  |
| 142 | C20 H32 O5 | 6.49 | 333.21 | Prostaglandin K1 | 1.09 | 1.50 | 5.22 | 0.11 | ↑## | ↓** |  |
| 143 | C28 H50 N O7 P | 8.69 | 544.34 | PC (18:4e/2:0) | 1.35 | 1.27 | 0.31 | 2.83 | ↓## | ↑** |  |
| 144 | C22 H32 O3 | 6.32 | 345.24 | Medroxyprogesterone | 1.31 | 1.34 | 5.56 | 0.19 | ↑## | ↓** |  |
| 145 | C27 H56 N O7 P | 10.55 | 538.39 | PC (16:0e/3:0) | 1.24 | 1.36 | 0.33 | 3.24 | ↓## | ↑** |  |
| 146 | C13 H16 N2 O5 | 5.29 | 281.11 | Asp-Phe | 1.14 | 1.39 | 0.59 | 1.88 | ↓## | ↑** |  |
| 147 | C14 H12 F3 N3 O3 | 4.76 | 653.16 | 3,5-dimethyl-N'-[4-(trifluoromethyl)benzoyl]isoxazole-4-carbohydrazide | 1.21 | 1.33 | 0.39 | 2.65 | ↓## | ↑** |  |
| 148 | C6 H8 O4 | 1.31 | 143.04 | trans-2-Butene-1,4-dicarboxylic Acid | 1.40 | 1.15 | 0.41 | 2.00 | ↓## | ↑** |  |
| 149 | C24 H40 O5 | 7.21 | 409.29 | Beta-Muricholic acid | 1.36 | 1.01 | 26.22 | 0.10 | ↑## | ↓** |  |
| 150 | C8 H12 O4 | 5.47 | 171.07 | 1,4-Cyclohexanedicarboxylic acid | 1.28 | 1.35 | 4.85 | 0.20 | ↑## | ↓** |  |
| 151 | C22 H46 N O7 P | 8.40 | 512.30 | LPC 14:0 | 1.03 | 1.50 | 0.48 | 2.71 | ↓## | ↑** |  |
| 152 | C24 H46 N O7 P | 8.55 | 492.31 | Lysopc 16:2 (2N Isomer) | 1.10 | 1.43 | 0.33 | 3.84 | ↓## | ↑** |  |
| 153 | C6 H13 N O3 S | 1.51 | 180.07 | Cyclohexylsulfamate | 1.11 | 1.38 | 0.39 | 3.02 | ↓## | ↑** |  |
| 154 | C20 H32 O4 | 7.70 | 337.24 | 8,15-Dihete | 1.35 | 1.21 | 1.98 | 0.56 | ↑## | ↓** |  |
| 155 | C30 H62 N O7 P | 11.81 | 624.43 | LPC 22:0 | 1.18 | 1.07 | 0.17 | 5.32 | ↓## | ↑* |  |
| 156 | C8 H8 O2 | 5.18 | 135.05 | o-Toluic Acid | 1.44 | 1.06 | 0.37 | 2.00 | ↓## | ↑** |  |
| 157 | C13 H22 O3 | 6.64 | 209.15 | 5-(6-hydroxy-6-methyloctyl)-2,5-dihydrofuran-2-one | 1.39 | 1.13 | 3.08 | 0.42 | ↑## | ↓** |  |
| 158 | C32 H58 N O7 P | 10.30 | 600.40 | PC (22:4e/2:0) | 1.20 | 1.40 | 0.45 | 2.46 | ↓## | ↑** |  |
| 159 | C16 H23 N5 O5 | 5.00 | 366.18 | Ip7G | 1.35 | 1.08 | 0.32 | 2.34 | ↓## | ↑** |  |
| 160 | C10 H18 O3 | 6.20 | 185.12 | Royal jelly acid | 1.08 | 1.40 | 0.42 | 3.08 | ↓## | ↑** |  |
| 161 | C26 H44 N Na O5 S | 6.21 | 504.28 | Taurolithocholic acid sodium salt | 1.32 | 1.39 | 15.57 | 0.06 | ↑## | ↓** |  |
| 162 | C6 H8 N2 O3 | 1.37 | 157.06 | L-beta-Imidazolelactic acid | 1.32 | 1.30 | 5.70 | 0.19 | ↑## | ↓** |  |
| 163 | C27 H42 N O4 | 7.30 | 444.32 | ACar 20:6 | 1.34 | 1.32 | 2.93 | 0.36 | ↑## | ↓** |  |
| 164 | C20 H32 O3 | 9.39 | 343.22 | 8(R)-Hydroxy-(5Z,9E,11Z,14Z)-eicosatetraenoic acid | 1.28 | 1.23 | 0.56 | 1.69 | ↓## | ↑** |  |
| 165 | C5 H8 O4 | 5.02 | 131.04 | Ethylmalonic acid | 1.37 | 1.05 | 0.47 | 1.75 | ↓## | ↑** |  |
| 166 | C21 H38 O4 | 9.21 | 355.28 | MAG (18:2) | 1.27 | 1.04 | 0.29 | 2.62 | ↓## | ↑** |  |
| 167 | C20 H25 N7 O6 | 4.87 | 460.19 | 5-Methyltetrahydrofolic acid | 1.12 | 1.48 | 0.37 | 3.54 | ↓## | ↑** |  |
| 168 | C22 H36 O4 | 8.75 | 387.25 | 16,16-Dimethyl prostaglandin A1 | 1.32 | 1.26 | 5.55 | 0.22 | ↑## | ↓** |  |
| 169 | C29 H38 O4 | 7.77 | 451.28 | Celastrol | 1.43 | 1.02 | 5.44 | 0.30 | ↑## | ↓** |  |
| 170 | C7 H6 O3 | 6.03 | 137.02 | 4-Hydroxybenzoic acid | 1.21 | 1.10 | 0.48 | 1.90 | ↓## | ↑** |  |
| 171 | C9 H16 O4 | 5.79 | 187.10 | Azelaic acid | 1.17 | 1.23 | 2.27 | 0.44 | ↑## | ↓** |  |
| 172 | C18 H34 O5 | 8.04 | 313.24 | (12Z)-9,10,11-trihydroxyoctadec-12-enoic acid | 1.09 | 1.30 | 2.28 | 0.39 | ↑## | ↓** |  |
| 173 | C14 H24 N2 S2 | 7.57 | 285.15 | N1,N2-dicyclohexylethanedithioamide | 1.05 | 1.51 | 0.17 | 12.60 | ↓## | ↑** |  |
| 174 | C40 H64 O4 | 10.19 | 607.47 | FAHFA (22:5/18:2) | 1.10 | 1.32 | 0.24 | 5.18 | ↓## | ↑** |  |
| 175 | C26 H44 O9 | 7.43 | 523.28 | Mupirocin | 1.10 | 1.41 | 5.91 | 0.10 | ↑## | ↓** |  |
| 176 | C19 H39 O7 P | 7.35 | 411.25 | Lysopa 16:0 | 1.34 | 1.24 | 9.17 | 0.15 | ↑## | ↓** |  |
| 177 | C20 H34 O2 | 10.20 | 307.26 | 11(Z),14(Z),17(Z)-Eicosatrienoic acid | 1.13 | 1.34 | 0.35 | 3.20 | ↓## | ↑** |  |
| 178 | C30 H50 N O8 P | 7.34 | 628.33 | PC (2:0/20:5) | 1.39 | 1.10 | 0.07 | 6.39 | ↓## | ↑** |  |
| 179 | C21 H32 O2 | 10.69 | 317.24 | 5α-Pregnan-3,20-dione | 1.08 | 1.36 | 0.42 | 2.92 | ↓## | ↑** |  |
| 180 | C20 H34 O6 | 6.27 | 369.23 | Thromboxane B2 | 1.10 | 1.18 | 0.46 | 2.27 | ↓## | ↑** |  |
| 181 | C27 H44 N O7 P | 8.78 | 524.28 | LPE 22:6 | 1.25 | 1.28 | 0.57 | 1.77 | ↓## | ↑** |  |
| 182 | C19 H28 O2 | 6.68 | 271.21 | Dehydroepiandrosterone (DHEA) | 1.36 | 1.01 | 19.74 | 0.14 | ↑## | ↓** |  |
| 183 | C25 H44 N O7 P | 8.83 | 500.28 | LPE 20:4 | 1.19 | 1.16 | 0.63 | 1.55 | ↓## | ↑** |  |
| 184 | C5 H6 O4 | 5.54 | 129.02 | Citraconic acid | 1.24 | 1.03 | 0.52 | 1.66 | ↓## | ↑** |  |
| 185 | C6 H8 O6 | 5.54 | 175.03 | L-Ascorbate | 1.24 | 1.01 | 0.52 | 1.65 | ↓## | ↑** |  |
| 186 | C21 H18 [2]H9 N O | 1.22 | 316.95 | methadone-d9 | 1.16 | 1.19 | 0.63 | 1.58 | ↓## | ↑** |  |
| 187 | C17 H15 Cl N2 O2 | 7.39 | 313.08 | N-benzyl-3-(4-chlorophenyl)-4,5-dihydro-5-isoxazolecarboxamide | 1.02 | 1.39 | 0.61 | 1.91 | ↓## | ↑** |  |
| 188 | C31 H43 N O7 | 7.10 | 542.31 | Milbemycin A3 oxime | 1.32 | 1.26 | 14.52 | 0.08 | ↑# | ↓* |  |
| 189 | C6 H12 O7 | 1.33 | 149.05 | Gluconic acid | 1.29 | 1.02 | 0.50 | 1.67 | ↓## | ↑** |  |
| 190 | C18 H34 O4 | 7.17 | 313.24 | (±)12(13)-DiHOME | 1.21 | 1.05 | 0.28 | 3.20 | ↓## | ↑** |  |
| 191 | C8 H15 N O3 | 5.61 | 172.10 | N-Acetyl-D-alloisoleucine | 1.20 | 1.07 | 0.41 | 2.16 | ↓## | ↑** |  |
| 192 | C26 H50 N O7 P | 8.95 | 518.32 | LPC 18:2 | 1.29 | 1.25 | 0.61 | 1.59 | ↓## | ↑** |  |
| 193 | C22 H23 [2]H5 N2 O | 6.11 | 342.26 | fentanyl-d5 | 1.08 | 1.18 | 2.26 | 0.43 | ↑## | ↓** |  |
| 194 | C23 H32 O6 | 7.52 | 403.21 | Hydrocortisone acetate | 1.01 | 1.42 | 0.46 | 2.89 | ↓## | ↑** |  |
| 195 | C30 H50 N O8 P | 7.34 | 584.33 | PC (4:0/18:5) | 1.34 | 1.18 | 0.10 | 5.79 | ↓## | ↑** |  |
| 196 | C32 H58 N O7 P | 10.31 | 644.40 | LPC 24:4 | 1.03 | 1.33 | 0.62 | 1.79 | ↓## | ↑** |  |
| 197 | C15 H24 N2 O2 | 7.68 | 265.19 | Oxymatrine | 1.31 | 1.14 | 0.13 | 4.91 | ↓## | ↑** |  |
| 198 | C6 H12 O3 | 5.63 | 131.07 | 2-Hydroxycaproic acid | 1.04 | 1.26 | 1.55 | 0.60 | ↑## | ↓** |  |
| 199 | C4 H9 N3 O2 | 1.36 | 132.08 | Creatine | 1.27 | 1.19 | 22.35 | 0.06 | ↑## | ↓** | B |
| 200 | C21 H32 O3 | 6.59 | 333.24 | 4-Pregnen-17alpha,20alpha-Diol-3-One | 1.42 | 1.41 | 1.21 | 0.56 | ↑# | ↓** |  |
| 201 | C20 H28 O | 8.64 | 285.22 | All trans-Retinal | 1.26 | 1.03 | 0.34 | 2.21 | ↓# | ↑** |  |
| 202 | C13 H14 N2 O4 | 5.27 | 263.10 | 2-(acetylamino)-3-[4-(acetylamino)phenyl]acrylic acid | 1.06 | 1.39 | 0.29 | 4.82 | ↓## | ↑** |  |
| 203 | C28 H50 N O8 P | 7.38 | 604.33 | PC (2:0/18:3) | 1.32 | 1.01 | 0.13 | 3.75 | ↓## | ↑** |  |
| 204 | C13 H16 O2 | 8.07 | 205.12 | 1-[4-hydroxy-3-(3-methylbut-2-en-1-yl)phenyl]ethan-1-one | 1.08 | 1.25 | 0.53 | 1.97 | ↓## | ↑** |  |
| 205 | C27 H52 N O7 P | 9.37 | 534.36 | PC (16:2e/3:0) | 1.23 | 1.21 | 0.28 | 3.22 | ↓## | ↑** |  |
| 206 | C28 H32 N2 O3 | 6.91 | 445.26 | 1-{4-[3-(4-benzhydrylpiperazino)-2-hydroxypropoxy]phenyl}ethan-1-one | 1.15 | 1.27 | 4.20 | 0.23 | ↑## | ↓** |  |
| 207 | C19 H39 O7 P | 6.28 | 427.25 | LPA 16:0 | 1.08 | 1.28 | 0.60 | 1.74 | ↓## | ↑** |  |
| 208 | C18 H34 O4 | 8.27 | 297.24 | (+/-)12(13)-DiHOME | 1.09 | 1.27 | 0.23 | 4.35 | ↓# | ↑** |  |
| 209 | C20 H32 O3 | 8.07 | 303.23 | (+/-)11(12)-EET | 1.05 | 1.21 | 0.36 | 3.13 | ↓## | ↑** | A |
| 210 | C11 H10 N2 O2 S | 1.25 | 235.06 | N-(6-methoxypyridin-3-yl)thiophene-2-carboxamide | 1.05 | 1.09 | 0.50 | 1.93 | ↓## | ↑** |  |
| 211 | C6 H14 N4 O2 | 1.27 | 175.12 | DL-Arginine | 1.10 | 1.12 | 0.27 | 3.70 | ↓## | ↑** |  |
| 212 | C20 H34 O6 | 7.91 | 371.24 | 6-Keto-prostaglandin f1alpha | 1.04 | 1.02 | 2.06 | 0.47 | ↑# | ↓** |  |
| 213 | C47 H93 N2 O6 P | 7.29 | 813.68 | SM (d30:1/12:1) | 1.02 | 1.13 | 3.50 | 0.27 | ↑ | ↓ |  |
| 214 | C48 H78 N O7 P | 7.89 | 812.55 | PC (20:5e/20:5) | 1.02 | 1.13 | 19.81 | 0.04 | ↑ | ↓ |  |
| 215 | C6 H6 N2 O | 1.86 | 123.06 | Nicotinamide | 1.25 | 1.04 | 2.56 | 0.47 | ↑## | ↓* | G |
| 216 | C20 H32 O6 | 6.53 | 413.22 | Prostaglandin G2 | 1.42 | 1.77 | 2.35 | 0.64 | ↑## | ↓** |  |
| 217 | C46 H79 O14 P | 7.11 | 885.51 | OxPG (18:0-22:6+4O) | 1.44 | 1.19 | 34.41 | 0.08 | ↑## | ↓** |  |
| 218 | C9 H9 N O2 | 5.29 | 164.07 | 7-Hydroxy-3,4-dihydrocarbostyril | 1.18 | 1.55 | 0.06 | 30.07 | ↓## | ↑** |  |
| 219 | C6 H12 O6 | 1.47 | 215.03 | Inositol | 1.15 | 1.19 | 0.12 | 6.27 | ↓ | ↑** | E |
| 220 | C28 H52 N O8 P | 7.54 | 606.34 | PC (4:0/16:2) | 1.38 | 1.56 | 0.45 | 1.32 | ↓## | ↑ |  |
| 221 | C28 H50 N O7 P | 8.87 | 588.33 | LPC 20:4 | 1.45 | 1.26 | 0.37 | 2.26 | ↓## | ↑** |  |
| 222 | C10 H16 N2 O3 S | 5.36 | 245.10 | Biotin | 1.02 | 1.14 | 0.48 | 2.32 | ↓## | ↑* | C |
| 223 | C19 H26 O3 | 7.32 | 325.18 | 2-Methoxyestradiol (2-MeOE2) | 1.65 | 1.57 | 0.57 | 3.87 | ↓## | ↑** |  |
| 224 | C19 H36 O4 | 9.09 | 329.27 | 1,4-dihydroxyheptadec-16-en-2-yl acetate | 1.31 | 1.41 | 0.11 | 9.27 | ↓## | ↑** |  |
| 225 | C24 H50 N O7 P | 9.29 | 494.33 | LPC 16:0 | 1.35 | 1.38 | 0.50 | 1.96 | ↓## | ↑** |  |
| 226 | C28 H54 N O7 P | 9.76 | 592.36 | LPC 20:2 | 1.39 | 1.29 | 0.27 | 3.17 | ↓## | ↑** |  |
| 227 | C23 H46 N O7 P | 8.64 | 478.30 | Lysope 18:1 | 1.14 | 1.53 | 0.33 | 4.09 | ↓## | ↑** |  |
| 228 | C11 H11 N O2 | 5.86 | 188.07 | Methyl 3-indolyacetate | 1.18 | 1.50 | 0.08 | 21.38 | ↓## | ↑** |  |
| 229 | C6 H13 N O3 | 1.43 | 130.09 | 4-Hydroxyisoleucine | 1.40 | 1.29 | 4.46 | 0.26 | ↑## | ↓** |  |
| 230 | C18 H36 O2 | 10.13 | 283.26 | Stearic Acid | 1.27 | 1.40 | 0.46 | 2.31 | ↓## | ↑** |  |
| 231 | C19 H24 N4 O | 7.85 | 347.18 | 1-(4-benzylpiperazino)-2-(pyridin-2-ylamino)propan-1-one | 1.40 | 1.27 | 5.74 | 0.22 | ↑## | ↓** |  |
| 232 | C18 H34 O2 | 8.89 | 327.25 | trans-Petroselinic Acid | 1.25 | 1.41 | 5.35 | 0.17 | ↑## | ↓** |  |
| 233 | C22 H46 N O7 P | 9.63 | 468.31 | LPE 17:0 | 1.12 | 1.35 | 0.56 | 2.00 | ↓## | ↑** |  |
